# Identification of the original plants of cultivated Bupleuri Radix based on DNA barcoding and chloroplast genome analysis

**DOI:** 10.7717/peerj.13208

**Published:** 2022-04-12

**Authors:** Gaixia Zhang, Hui Wang, Linchun Shi, Yang Liu, Ruyu Yao, Chun Sui, Chengmin Yang, Hongliang Ji, Qiuling Wang, Jianhe Wei

**Affiliations:** 1 Institute of Medicinal Plant Development, Chinese Academy of Medical Sciences and Peking Union Medical College, Beijing, China; 2 Institute of Sericulture, Chengde Medical University, Chengde, China

**Keywords:** Cultivated *Bupleurum*, Identification, DNA barcoding, Chloroplast genome, New DNA markers

## Abstract

Bupleuri Radix is the dry root of certain species of the genus *Bupleurum* and is commonly used in traditional Chinese medicine. The increasing global demand for Bupleuri Radix cannot be fulfilled with wild populations only. Therefore, cultivated *Bupleurum* is now the main commercial source of this medicinal product. Different species of *Bupleurum* show different medicinal properties and clinical effects, making reliable authentication and assignment of correct botanical origin for medicinal species critical. However, accurate identification of the cultivated *Bupleurum* species is difficult due to dramatic morphological variations resulting from cultivation. In this study, we sampled 56 cultivated *Bupleurum* populations of six different morphotypes (Types A-F) from the main production areas of China, and 10 wild populations of four species were used as reference materials. Conventional DNA barcoding was conducted to identify cultivated *Bupleurum* species. Additionally, verification based on complete chloroplast genomes was performed and new chloroplast markers were developed and evaluated. The combination of these methods resulted in the successful identification of all cultivated *Bupleurum* individuals. Three chloroplast regions are recommended as additional barcodes for the genus: *ycf4_cemA, psaJ_rpl33*, and *ndhE_ndhG*. This is a reliable and promising strategy that can be applied to the authentication of natural products and the identification of other medicinal plant species with similar taxonomic problems.

## Introduction

As a plant with high diversity, Bupleuri Radix has been widely used as a herbal drug in Asia over the past 2,000 years and is famous for the treatment of various diseases such as typhoid fever, malaria, epidemic colds, hepatitis, menstrual irregularities, and pain from swollen breasts, uterine prolapse, and prolapse of the rectum (Chinese Pharmacopoeia Commission, 2015; Gorovoy, Ketrits & Grief, 1980; Pan, 2006; Young Hwa et al., 2012; Yuan, Li & Ma, 2017a). It has also been recommended to treat COVID-19 with other traditional Chinese medicine (Fan et al., 2020; Sun et al., 2020; Zhou & Wang, 2020). The original herbs of Bupleuri Radix belong to *Bupleurum* L., which is a primitive and large genera of the family Apiaceae (Umbelliferae) (Wang et al., 2008). *Bupleurum* L. is widely distributed throughout the North Temperate Zone and consists of around 180 species, of which 44 species, 17 varieties, and 7 forma have been reported in China (Pan et al, 2002; She & Watson, 2005). Some studies suggested that the Chinese *Bupleurum* species are divided into two major lineages and should be placed in Neves and Watson’s subgenus *Bupleurum* (Wang, Ma & He, 2011). Given the conflicting opinions about the taxonomic rank of most *Bupleurum* taxa and the dramatic morphological variations within species, the taxonomy of the group is complex, and several species remain difficult to identify (Chao et al., 2014; Pan, 2006; She & Watson, 2005; Wang, Ma & He, 2011; Wang et al., 2008). Furthermore, wild populations of *Bupleurum* are not sufficient to meet the current market demand for Bupleuri Radix. Thus, cultivated species are now the main commercial source of this medicinal herb because they produce a stable supply and considerable yield (Liang, 2012; Liang, Liu & Chao, 2012; Liu et al., 2011; Minami et al., 1997; Qin et al., 2012; Shimokawa & Oiiashi, 1980; Shon, Haryanto & Yoshida, 1997; Zhao et al., 2011; Zhu et al., 2017).

In China, *Bupleurum* species are extensively cultivated in the provinces of Gansu, Shaanxi, Shanxi, Heilongjiang, and Hebei. In our previous study (Zhang et al., 2021), through observation and systematic comparison of living plants and specimens of *Bupleurum* spp., we found that the cultivated *Bupleurum* spp. could be preliminarily divided into six phenotypes (Types A-F) and ascribed to four species (*B. chinense* DC., *B. scorzonerifolium* Willd., *B. falcatum* Linneus, and *B. marginatum* var. *stenophyllum* (Wolff) Shan et Y. Li) based on 13 distinguishable morphological characteristics, including root color, growth pattern of basal leaf phyllotaxis and rhizome buds, roughness of stem surface, and fruit length. During the previous investigation, we found that morphological variations and the lack of reliable identification methods of cultivated *Bupleurum* populations have made it increasingly difficult to accurately delimit species. The same *Bupleurum* plant may be considered as different species due to morphological variations and/or the employment of mislabeled sequences into the identification analysis. For example, the same cultivated germplasm of Bupleuri Radix from Gansu Province was identified as three different species (*B. chinense* DC., *B. yinchowense* Shan et Y. Li, or *B. marginatum* Wall. ex DC.) in various studies (Geng et al., 2010; Guo et al., 2018; Liang, 2012; Qin et al., 2012; Yang et al., 2019; Zhu et al., 2017). Two morphotypes produced in Heilongjiang Province were temporarily treated as the same germplasm and its botanical origin could not be determined (Du et al., 2019). Accurate species identification within *Bupleurum* is critical because the pharmacological value and clinical properties vary between species and they should not be used interchangeably (Ashour et al., 2009; Huang et al., 2009; Lee et al., 2011; Li et al., 2007; Li et al., 2005; Liu et al., 2011; Qin et al., 2012). Though *B. chinense* and *B. scorzonerifolium* Willd. are regarded as standard medical plants of Bupleuri Radix in the Pharmacopeia of the People’s Republic of China (Chinese Pharmacopoeia Commission, 2015), many species recorded in the literature are regional substitutes for Bupleuri Radix or for other medicinal uses, some of them are even with toxicity. For example, it has been reported that *B. longiradiatum* Turcz. is toxic and cannot be used as a source of Bupleuri Radix (Ashour & Wink, 2011; Lin, Zhang & Su, 2016). The safe use of this medicinal plant and its derived products urgently demand the development of specific and accurate methods to effectively determine the species that make Bupleuri Radix.

Here, the DNA barcoding technology was applied to further identify the cultivated germplasm of Bupleuri Radix at the species level. The phylogenetic analysis using chloroplast genomes and molecular markers developed from chloroplast genome sequences were introduced to evaluate and validate the identification result based on DNA barcodes. As an efficient tool for the authentication of medicinal plants and herbal materials, the DNA barcoding technology has been widely applied for species identification (Chen et al., 2014; Song et al., 2009). Chloroplast genomes are a useful tool for phylogenetic analyses and comparative studies, and as a source of alternative DNA markers because they are highly conserved with respect to their genome size, structure, and gene content (Asaf et al., 2017; Chen et al., 2018; Jiang et al., 2017). Additional DNA markers were harvested from complete chloroplast genomes and evaluated for species that could not be fully identified using DNA barcoding alone.

## Materials & Methods

### Materials

Leaves for all six morphotypes (Types A-F) were freshly collected in triplicates from 56 cultivated populations from the main Bupleuri Radix production areas of China. Four wild species (*B. falcatum*, *B. scorzonerifolium*, *B. marginatum* var. *stenophyllum* (Wolff) Shan et Y.Li, and *B. chinense*) corresponding to cultivated species or with ambiguous phylogenetic classifications to cultivated species were collected and used as the reference material for cultivated species identification (Table 1, Table S1). Herbarium vouchers for both cultivated and wild specimens were deposited in the Institute of Medicinal Plant Development (IMPLAD).

**Table 1:** Population information of *Bupleurum* used in this study.

Serial No. of populations	Morphological type/species	Origin	Producing area
HLC01-HLC02, HLC05	A	Cultivated	Heilongjiang
HEC01-HEC03	A	Cultivated	Hebei
HLC03-HLC04, HLC06	B	Cultivated	Heilongjiang
GSC01- GSC05	C	Cultivated	Gansu
GSC06- GSC18	D	Cultivated	Gansu
HEC04-HEC06	D	Cultivated	Hebei
SXC08	D	Cultivated	Shanxi
SXC01-SXC07, SXC09-SXC12	E	Cultivated	Shanxi
SNC01-SNC06	E	Cultivated	Shaanxi
SNC07-SNC14	F	Cultivated	Shaanxi
GSW01-GSW02	*B. chinense*	Wild	Gansu
HLW01	*B. falcatum*	Wild	Heilongjiang
HLW02	*B. scorzonerifolium*	Wild	Heilongjiang
HEW01	*B. scorzonerifolium*	Wild	Hebei
SXW01	*B. chinense*	Wild	Shanxi
SNW01-SNW03	*B. chinense*	Wild	Shaanxi
XZW01	*B. marginatum* var. *stenophyllum*	Wild	Xizang

### DNA barcoding analysis

Four conventional DNA barcodes (internal transcribed spacer - ITS, *psbA*-*trnH*, *rbcL*, and *matK*) were initially tested for their ability to discriminate cultivated and wild specimens. The primers used for amplification were as previously reported (Chen, 2012). A preliminary survey to assess barcode suitability was done with 63 samples from 47 cultivated populations representing six morphotypes. Meanwhile, 19 samples from seven wild populations were selected as reference material for the identification of cultivated species (Tables S1, S2). DNA extraction, PCR amplification, sequencing and sequence alignment were performed according to previously published procedures (Han et al., 2013; Song et al., 2009). Analysis of sequence variation among the cultivated species and the reference species was performed using the Molecular Evolutionary Genetics Analysis (MEGA) software (Kumar et al., 2018). A phylogenetic tree was constructed using the neighbor-joining algorithm (NJ tree) with 1,000 bootstrap replicates. Once the most suitable barcode (ITS) was selected, we expanded our sample set to further include 36 cultivated individuals (Tables S1, S2) and 11 wild individuals (Tables S1, S2). As a result, the ITS was examined in a total of 99 cultivated samples from 56 populations and 30 wild samples from 10 populations. ITS sequences of *Angelica sinensis* (JN704870) and *Hansenia forbesii* (JQ936553) were obtained from GenBank and used as outgroups for the NJ tree.

### Verification based on complete chloroplast genomes

DNA extraction, sequencing, and annotation of chloroplast genomes were conducted as per Chen et al. (2018) and Zhou et al. (2018). Chloroplast sequence cluster analysis was performed on seven representative cultivated samples (6 cultivation morphotypes represented by HEC02-3, HLC04-3, GSC03-1, GSC06-1, SXC04-1, SNC10-1, and 1 adulterant germplasm of cultivated *B. scorzonerifolium* represented by HLC05-3). The chloroplast genomes of *B. falcatum* (HEC02-3, MT075714; HLC05-3, MT075716), *B. chinense* (GSC06-1, MT075713; SXC04-1, MT075710; SNC10-1, MT075709), and *B. scorzonerifolium* (HLC04-3, MT075715) are newly generated in this research, and the chloroplast genomes of GSC03-1(MT075712) was obtained from my previous research (Zhang et al., 2021). Similarities between the chloroplast genomes of the seven samples were calculated as described by Park et al. (2018).

To determine the phylogenetic relationship and genetic distance between morphotypes and each species, chloroplast genome sequences for *B. chinense* (NC_046774, MN893666), *B. scorzonerifolium* (MT239475), *B. falcatum* (NC_027834, MT821947), *B. marginatum* (MN968501), *B. latissimum* (NC_033346, MT821949), *Angelica sinensis* (MH430891), and *Hansenia forbesii* (NC_035054) (outgroup), were obtained from GenBank. Firstly, using MAFFT(v7.309) (Katoh & Standley, 2013), complete genome alignments were generated as well as with 74 genes shared by the 17 genome sequences. MrModeltest (v2.4) (Nylander, 2004) was then used to determine the best-fitting model based on the Akaike Information Criterion, and the optimal model, GTR+I+G, was selected for both datasets. Maximum likelihood (ML) analysis was performed using RaxML (v8.2.12) (Stamatakis, 2014) with 1000 bootstrap replicates. Bayesian Inference (BI) analysis was performed using MrBayes (v3.2.7) (Ronquist et al., 2012). Markov Chain Monte Carlo simulations for 2,000,000 generations were independently performed twice, sampling every 100th generation. Convergence was determined by examining the average standard deviation of split frequencies (<0.01). The first 25% of trees were discarded as burn-in, and the remaining trees were used to build a majority-rule consensus tree. Maximum parsimony (MP) analysis was run in Paup (v4.0b10) (Swofford, 2003), using heuristic search and tree bisection-reconnection (TBR) branch swapping with 1000 bootstrap replicates. Neighbor-Joining (NJ) analysis and genetic distance calculation were conducted using MEGA X (Kumar et al., 2018).

### Development and validation of additional chloroplast DNA markers

In order to select a short and informative region with enough variation, nucleotide variability (Pi) was calculated for both coding and non-coding regions of the chloroplast using DnaSP version 5.1 (Librado & Rozas, 2009). Highly variable regions with Pi values greater than or equal to 0.015 and with high discriminatory power were screened as potential barcodes through extraction, aligned using MUSCLE, and analysed using the neighbor-joining algorithm (NJ tree) of sequences. Primers were designed using Primer Premier 5.0. PCR amplification was performed in a 25-µl reaction as follows: initial denaturation at 94 °C for 5 min; 40 cycles at 94 °C for 30 s, 56 °C for 30s, and 72 °C for 45 s; and final extension at 72 °C for 10 min. The PCR products were sequenced on an ABI 3730 sequence analyzer (Applied Biosystems Inc., CA, United States) with the same primers used for PCR amplification. The 21 samples used for marker verification are listed in Table S3. All primers for marker selection are shown in Table S4. The identification efficiency of potential markers was evaluated as described in ‘DNA Barcoding Analysis’.

## Results

### DNA barcoding identification

**Figure 1:**
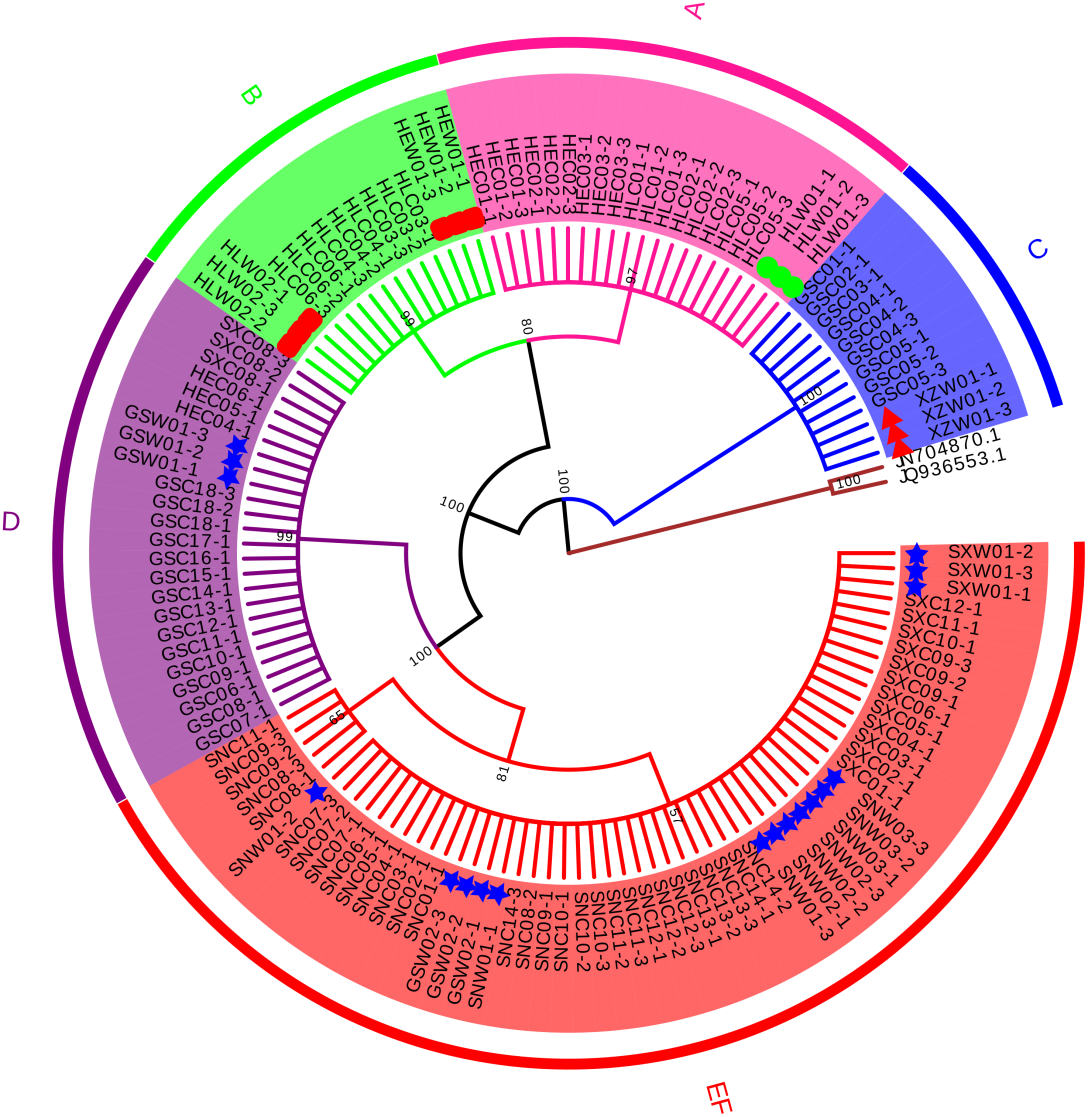
Neighbor Joining tree based on ITS sequences of cultivated *Bupleurum* types and associated reference species. Bootstrap support values are shown on each branch. Type A samples are highlighted in pink, Type B in green, Type C in blue, Type D in purple, and Types E and F in red. Green circles indicate reference specimens of *B. falcatum*, red squares indicate reference specimens of *B. scorzonerifolium*, red triangles indicate reference specimens of *B. marginatum* var. *stenophyllum*, blue stars indicate reference specimens of *B. chinense*.

Four conventional DNA barcodes (ITS, *psbA-trnH*, *rbcL*, and *matK*) were tested to evaluate their identification efficiency. Since *psbA-trnH* is a non-coding region, it is rich in long indels and poly (dA) and poly (dT), these sequence features will interfere with sequencing results (Fig. S1, Table S5). Therefore, *psbA*-*trnH* is not suitable for species identification. *matK* and *rbcL* were not variable enough to discriminate morphotypes or species (Figs. S2–S3, Tables S6–S7). ITS, on the other hand, showed effective discriminatory power and was selected to confirm species identification of the cultivated samples (Fig. 1, Table 2). A total of 129 ITS sequences were obtained: 30 wild specimens of *B. chinense*, *B. scorzonerifolium*, *B. falcatum*, and *B. marginatum* var. *stenophyllum*, and 99 cultivated samples of all six morphotypes (24 from Gansu Province, 18 from Heilongjiang Province, 12 from Hebei Province, 15 from Shanxi Province, and 30 from Shaanxi Province). Sequence length before alignment was 603–609 bp. No variability was observed within the phenotypes, with the exception of Type F, which had one variable site (420 T-C) and was therefore divided into two haplotypes (Table 2). Types A, B, and C were identified as *B. falcatum* (HLW01), *B. scorzonerifolium* (HEW01; HLW02), and *B. marginatum* var. *stenophyllum* (XZW01), respectively, and Types D, E and F as *B. chinense* , Type D, Types E and F matched *B. chinense* (GSW01), *B. chinense* (SXW01, SNW01-1, SNW01-3; GSW02; SNW03, SNW02), respectively (Fig. 1, Table 2), and the three Tpyes were grouped into a single, separate clade (Fig. 1).

**Table 2:** Variable sites in the haplotypes of different cultivated *Bupleurum* types.

	0000000000	0000000001	1111111111	1111111122	2333444444	4444455555	5555555555	5666
Haplotype	0111344445	6677777890	0111122255	7888899901	1588011122	2268802333	3556777888	8001
	5789813465	0424789924	8356978989	6156927924	9337212501	2747919126	7071235023	4587
Type A	AAAGTAGAGG	TCTAGCTACT	TCGCGACCCC	TGCGTGGTAG	ACTTAAACTC	ATCTTAGATG	CCTCCCTACA	GATG
Type B	.........T	....T.....	..........	..........	..A..G....	G..C......	.....T...T	....
Type C	GCGAA.TGT.	CT.GATGGT.	CTCT.TATTT	CCGAGACG.T	CTAATGTT.T	GAT..T..CT	A.CTA.CG..	.CCA
Type D	.....T....	..C......C	....A.....	C.........	..AA......	G...A.TG..	.A...T..A.	T..A
Type E	..........	C.C......C	..........	C.........	..AA......	G...A.TG..	.A...T..A.	T...
Type F1	..........	C.C......C	..........	C.........	..AA......	G...A.TG..	.A...T..A.	T...
Type F2^∗^	..........	C.C......C	..........	C.........	..AA....C.	G...A.TG..	.A...T..A.	T...

**Notes.**

* Type F has two haplotypes; SNC08-1, SNC08-3, SNC09-2, SNC09-3, and SNC11-1 belong to haplotype F2, and the remaining sequences are consistent with the dominant F1 haplotype.

### Verification based on chloroplast genomes

**Figure 2:**
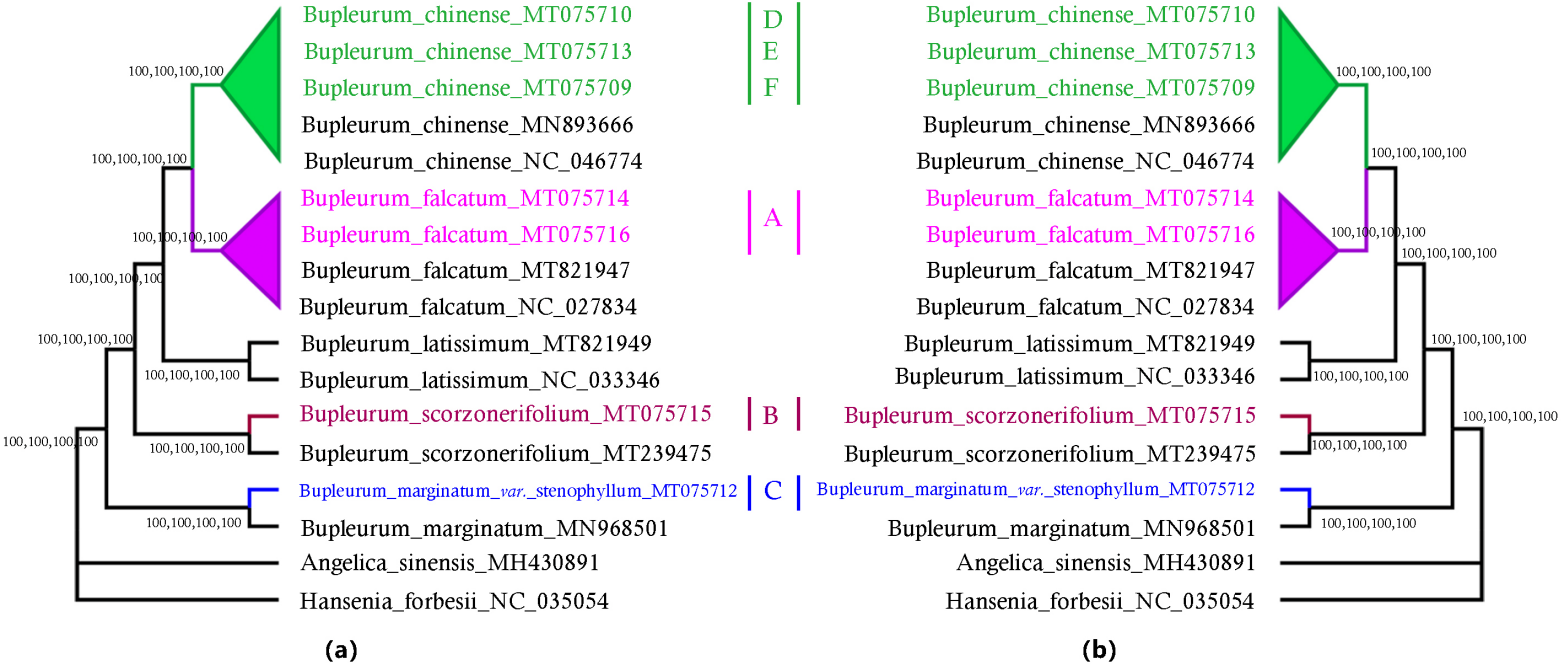
MP, ML, NJ, and BI phylogenetic trees for cultivated types and reference species of the genus *Bupleurum* using (A) 74 chloroplast genes and, (B) the entire chloroplast genome. Numbers above the branches are support values of Maximum Parsimony, Maximum Likelihood, Neighbor Joining and Bayesian Inference, respectively. The subtree labels A, B, C, D, E, and F indicate representative samples of Type A, Type B, Type C, Type D, Type E, and Type F, respectively.

A total of 79 protein-coding genes were annotated in the chloroplast genome of the studied *Bupleurum* species. Complete chloroplast genomes and all the 74 genes shared among 17 members of the genus *Bupleurum* and two other species within the family Umbelliferae (*A. sinensis* and *H. forbesii*) were analyzed. Bayesian inference (BI), maximum parsimony (MP), Neighbor-Joining (NJ), and maximum likelihood (ML) generated identical tree topologies for the main clades (Fig. 2 and Figs. S4–S11).

Among the species having been identified by morphological characteristics and DNA barcodes, Type A samples from Heilongjiang (HLC05-3, MT075716) and Hebei Provinces (HEC02-3, MT075714) corresponded to the reference chloroplast genome of *B. falcatum* (NC_027834, MT821947). Type B samples (MT075715) corresponded to the reference chloroplast genome of *B. scorzonerifolium* (MT239475). *B. marginatum* var. *stenophyllum* (MT075712) was closely clustered with *B. marginatum* (MN968501) and possessed a basal position sister to all the other *Bupleurum* species. Type D was clustered in the same clade with *B. chinense* (Type E, MT075710; Type F, MT075709) and corresponded to the reference chloroplast genome of *B. chinense* (NC_046774; MN893666). Support values of the species clades were high (100) and intraspecific support values varied between the phylogenetic tree constructed using the complete chloroplast genomes and that constructed using the shared genes (Figs. S4–S11). Furthermore, the maximum intraspecific genetic distance within each species was lower than the corresponding minimum interspecific genetic distance (Table S9), which confirmed the reliability of the identification results obtained from morphological characteristics and DNA barcodes.

### Highly Variable Chloroplast Regions for the Development of New DNA Markers

**Figure 3:**
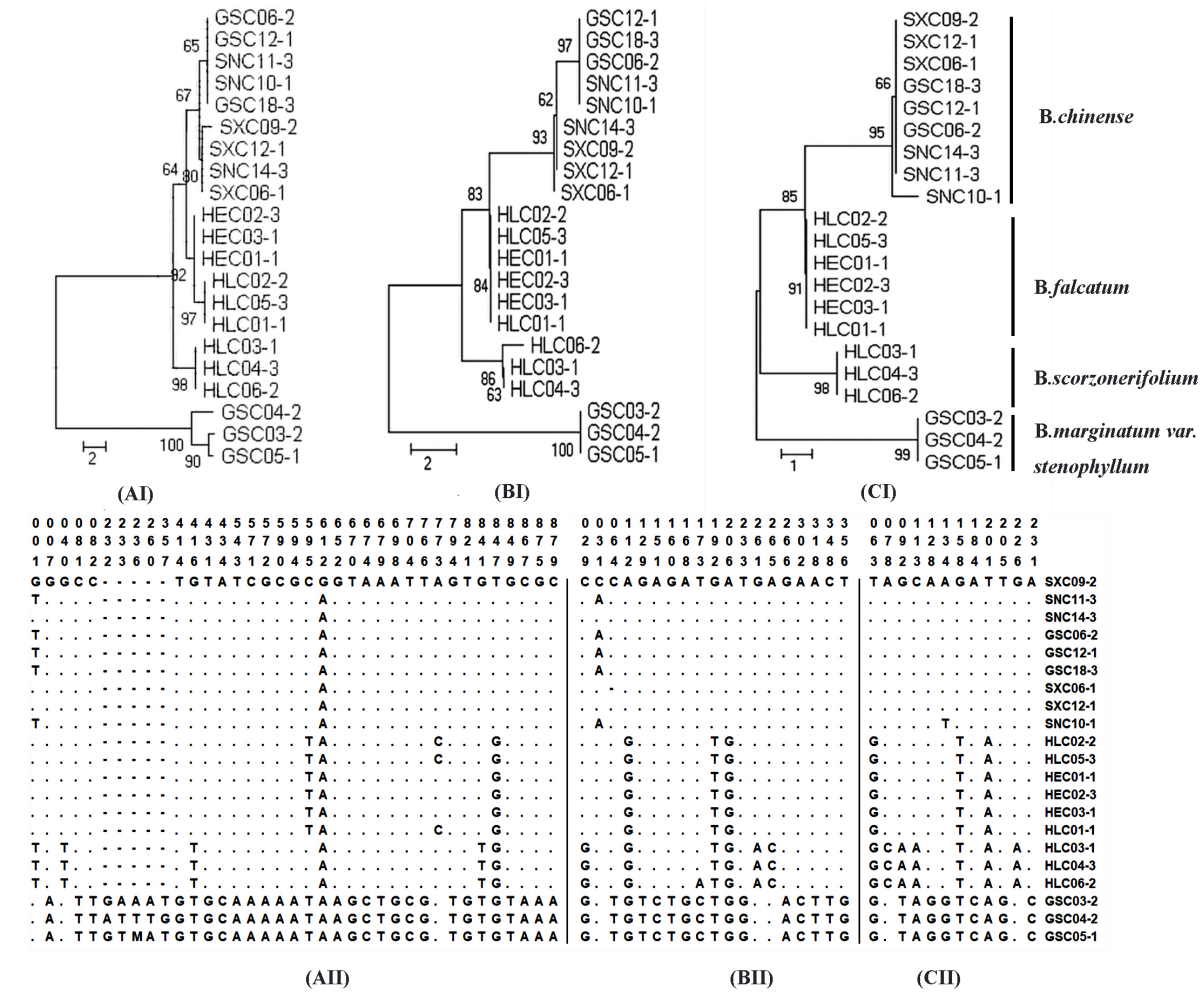
Analysis of neighbor-joining phylogenetic tree and variable sites for four cultivated species of the genus *Bupleurum* based on *ycf4_cemA* (AI, AII) *psaJ_rpl33* (BI, BII), and *ndhE_ndhG* (CI, CII) sequences. No. of differences is selected as the analysis preference of the model for the purpose of species identification. The bootstrap values (1000 replicates) are shown for each branch.

Sequence divergence was further analyzed by extracting coding and non-coding regions from the chloroplast genomes sequences to calculate nucleotide variability (Pi) (Table S10–S11). Pi values ranged from 0 to 0.0433. Non-coding regions were more variable compared with the coding regions. Nineteen regions with nucleotide diversity >0.015 were selected and assessed through sequence variation analysis and phylogenetic analysis. The ideal DNA marker should be short enough for easy PCR amplification and sequencing, have sufficient interspecific variation but low intraspecific variation, and have conservative flanking sequences for easy primer design. Based on these considerations, three DNA markers (*ycf4_cemA*, *psaJ_rpl33*, and *ndhE_ndhG*), which were verified by conventional DNA barcoding methods to successfully discriminate cultivated *Bupleurum*, were selected and recommended as complementary barcodes for *Bupleurum* identification. Detailed results of the sequence variation and phylogenetic analysis are shown in Fig. 3.

## Discussion

### Accurate identification of cultivated *Bupleurum* species in China

The conventional DNA barcoding technology was conducted to identify cultivated *Bupleurum* species, the complete chloroplast genomes were used to verify the identification results obtained with DNA barcodes, and DNA markers developed from chloroplast genome sequences were introduced to further evaluate and validate the results of previous identifications. The combination of these three methods successfully determined the species identity of cultivated *Bupleurum* in China, including *B. chinense*, produced mainly in Gansu Province, and *B. falcatum*, produced in Heilongjiang Province (Ding et al., 2016; Du et al., 2019; Geng et al., 2010; Guo et al., 2018; Qin et al., 2012; Yang et al., 2019; Yuan et al., 2017b; Zhu et al., 2017). Former studies were unable to determine the species identity for germplasm from Gansu Province. Three possible identifications have been proposed: *B. chinense*, *B. yinchowense*, and *B. marginatum* (Chao et al., 2014; Ding et al., 2016; Wang, Ma & He, 2011; Wang et al., 2008; Xie et al., 2009; Yang et al., 2007; Yuan et al., 2017b). Firstly, our previous morphological analysis supports the attribution of *B. chinense* based on morphological character descriptions published in Flora of China and the distinguishable morphological characteristics from our analysis and summary. The verification results based on ITS sequences and chloroplast genome analysis and the newly developed markers in the present study all supported its attribution to *B. chinense*.

Previous studies have treated cultivated *B. falcatum* as an adulterant of *B. scorzonerifolium* produced in Heilongjiang Province, which was temporarily treated as a morphotype of *B. scorzonerifolium* (Du et al., 2019). Our results indicate that *B. scorzonerifolium* adulterants from the Heilongjiang and Hebei Provinces are the same species, and were identified as *B. falcatum*. Equal chromosome number and closer genome size are congruent with this conclusion (Du et al., 2019). *B. falcatum* from China has been considered as the same species in Japan and Korea (Gorovoy, Ketrits & Grief, 1980; Jiang, Xu & Li, 2000; Jiang et al., 1994; Li et al., 1994; Matsumoto et al., 2004; Pan et al., 1995; Wang, 2011; Wang, Ma & He, 2011; Wang, Ma & He, 2013; Wang et al., 2016a), but it has not been included in the Flora of China. Considering its wide distribution and abundance (Jiang, Xu & Li, 2000), we suggest that *B. falcatum* should be included in the Flora of China, which would facilitate and encourage its medicinal use.

### Identification methods for cultivated *Bupleurum* and potential applications

Natural foods and medicines have become increasingly popular in recent years due to growing public awareness about nutrition and health issues (Phan, David & Sabaratnam, 2017; Xin et al., 2013; Yao et al., 2018). To ensure their appropriate, safe, and effective use, a precise and clear species identification of these products is paramount. Many plant species have similar taxonomic classification problems that result from domestication. For example, yams (*Dioscorea* spp.) are an important food crop with significant medicinal effects for spleen deficiency, reduced food intake, chronic diarrhea, etc. However, the taxonomy of the group is complex and remains unresolved because of the great variation resulting from domestication and artificial breeding (Gao et al., 2008; Wu, 2012). Similar issues are encountered in other medicinal crops such as mulberry (*Morus* spp.) and Goji (fruits of *Lycium barbarum* L. and *L. chinense* Mill.) (Gao et al., 2015; Xin et al., 2013; Yin, 2013; Zeng et al., 2015). Cultivated *Bupleurum* individuals were identified at the species level using DNA barcodes and further verified by phylogenetic analyses of complete chloroplast genomes and newly developed markers. The methods applied in this study provide a possible solution for these challenges and may serve as a powerful tool to solve taxonomic problems and ensure quality control of medicinal plants.

Our results confirmed that the relatively less sequence variations in conventional chloroplast barcodes (*i.e., rbcL*, *matK*, and *trnH-psbA*) among *Bupleurum* species might lead to incorrect identification result at the inter-generic level (Tables S5–S7, Figs. S1–S3). However, complete chloroplast genome analyses did provide enough discriminatory power to identify all species and morphotypes. Since the use of chloroplast genomes is not applicable to all sample types (*e.g.*, degraded and processed samples with low DNA concentration and quality) and available to all research groups, we selected the three most variable chloroplast regions and recommend their use for species identification in *Bupleurum* to complement ITS: *ycf4_cemA*, *psaJ_rpl33*, and *ndhE_ndhG*. These markers can be used to streamline the identification of degraded and processed samples, and to facilitate and expedite the identification of *Bupleurum* species at a reduced cost: *ycf4_cemA*, *psaJ_rpl33*, and *ndhE_ndhG.* In future studies, we will include more species or samples to further exert the identification effectiveness of complete chloroplast genomes and expand the application of the developed markers on crude drugs of *Bupleurum* species as well as their products.

## Conclusions

We presented an identification pipeline to accurately and specifically identify cultivated species of *Bupleurum* in China. This approach combines DNA barcoding, chloroplast genomes, and genus specific markers (*ycf4_cemA*, *psaJ_rpl33*, and *ndhE_ndhG*), and provides multiple and independent evidence to verify species identity. It also improves the efficiency and accuracy for the identification of cultivated *Bupleurum* species, which is critical for the development of resources that can be used in natural products, and for the safe and effective use of Bupleuri Radix. The combination of these methods could be equally successful to address similar taxonomic problems in other plant groups.

##  Supplemental Information

10.7717/peerj.13208/supp-1 Supplemental Information 1 Neighbor-joining phylogenetic tree constructed based on the *psbA-trnH* sequences of cultivated *Bupleurum* types and reference species The bootstrap scores (1000 replicates) are shown for each branch.

10.7717/peerj.13208/supp-2 Supplemental Information 2 Neighbor-joining phylogenetic tree constructed based on the *matK* sequences of cultivated *Bupleurum* types and reference species The bootstrap scores (1000 replicates) are shown for each branch.

10.7717/peerj.13208/supp-3 Supplemental Information 3 Neighbor-joining phylogenetic tree constructed based on the *rbcL* sequences of cultivated *Bupleurum* types and reference species The bootstrap scores (1000 replicates) are shown for each branch.

10.7717/peerj.13208/supp-4 Supplemental Information 4 Phylogenetic trees of the entire genome sequences from cultivated types and reference species constructed using the Bayesian inference method

10.7717/peerj.13208/supp-5 Supplemental Information 5 Phylogenetic trees of the entire genome sequences from cultivated types and reference species constructed using the maximum parsimony method

10.7717/peerj.13208/supp-6 Supplemental Information 6 Phylogenetic trees of the entire genome sequences from cultivated types and reference species constructed using the maximum likelihood method

10.7717/peerj.13208/supp-7 Supplemental Information 7 Phylogenetic trees of the entire genome sequences from cultivated types and reference species constructed using the neighbor-joining method

10.7717/peerj.13208/supp-8 Supplemental Information 8 Phylogenetic trees of the shared chloroplast gene sequences from cultivated types and reference species constructed using the Bayesian inference method

10.7717/peerj.13208/supp-9 Supplemental Information 9 Phylogenetic trees of the shared chloroplast gene sequences from cultivated types and reference species constructed using the maximum likelihood method

10.7717/peerj.13208/supp-10 Supplemental Information 10 Phylogenetic trees of the shared chloroplast gene sequences from cultivated types and reference species constructed using the maximum parsimony method

10.7717/peerj.13208/supp-11 Supplemental Information 11 Phylogenetic trees of the shared chloroplast gene sequences from cultivated types and reference species constructed using the neighbor-joining method

10.7717/peerj.13208/supp-12 Supplemental Information 12 *Bupleurum* populations sampled in this study

10.7717/peerj.13208/supp-13 Supplemental Information 13 GenBank accession numbers for the DNA barcodes generated in this study - indicates that there is no corresponding experiment for the sample.

10.7717/peerj.13208/supp-14 Supplemental Information 14 Cultivated *Bupleurum* samples used for barcode validation

10.7717/peerj.13208/supp-15 Supplemental Information 15 PCR primers for the 19 most variable regions among the seven *Bupleurum* chloroplast genome sequences

10.7717/peerj.13208/supp-16 Supplemental Information 16 Variable sites of *psbA-trnH* sequences in the samples of different cultivated *Bupleurum* types and reference species

10.7717/peerj.13208/supp-17 Supplemental Information 17 Variable sites of *matK* sequences in the samples of cultivated *Bupleurum* types and reference species

10.7717/peerj.13208/supp-18 Supplemental Information 18 Variable sites of *rbcL* sequences in the samples of the different cultivated *Bupleurum* types and reference species

10.7717/peerj.13208/supp-19 Supplemental Information 19 Sequence similarities between the chloroplast genomes of cultivated *Bupleurum* types

10.7717/peerj.13208/supp-20 Supplemental Information 20 Genetic distance among cultivated *Bupleurum* types and species based on complete chloroplast genome sequences

10.7717/peerj.13208/supp-21 Supplemental Information 21 Nucleotide diversity values of the noncoding regions among the seven *Bupleurum* chloroplast genome sequences

10.7717/peerj.13208/supp-22 Supplemental Information 22 Nucleotide diversity values of the coding regions among the seven *Bupleurum* chloroplast genome sequences

10.7717/peerj.13208/supp-23 Supplemental Information 23 Raw data

## Additional Information and Declarations

### Field Study Permissions

The following information was supplied relating to field study approvals (i.e., approving body and any reference numbers): The field materials were belong to individual farmers, the materials used in this study were obtained with verbal permission from local farmers of every collection point. For farmers who are not willing to disclose their personal information, we have written the informations of the guides. The relevant informations have been provided as a confidential supplemental file named “Field Permit”. Thank you.

### Data Availability

The following information was supplied regarding data availability: The raw data are available in the Supplemental Files and at Genbank: ITS (MT074485–MT074610, MT074614–MT074616); psbA-trnH (MT085836–MT085848, MT085850–MT085866, MT085869–MT085880, MT085883–MT085886, MT085888–MT085902, MT085905–MT08590217, MT085920–MT085925); matK (MT085926–MT085974, MT085976–MT085989, MT085991–MT085996, MT085998–MT086011); rbcL (MT086018–MT086021, MT086023, MT086025–MT086056, MT086058–MT086063, MT086065–MT086071, MT086074–MT086077, MT086079–MT086085, MT086089–MT086096, MT086098–MT086110); Chloroplast genome (MT075709, MT075710, MT075712–MT075716).
