# Supplementary figures and images for: Identification of the original plants of cultivated Bupleuri Radix based on DNA barcoding and chloroplast genome analysis
Source: PeerJ. 2022 Apr 12;10:e13208. doi: 10.7717/peerj.13208 (PMC9012172; doi:10.7717/peerj.13208)

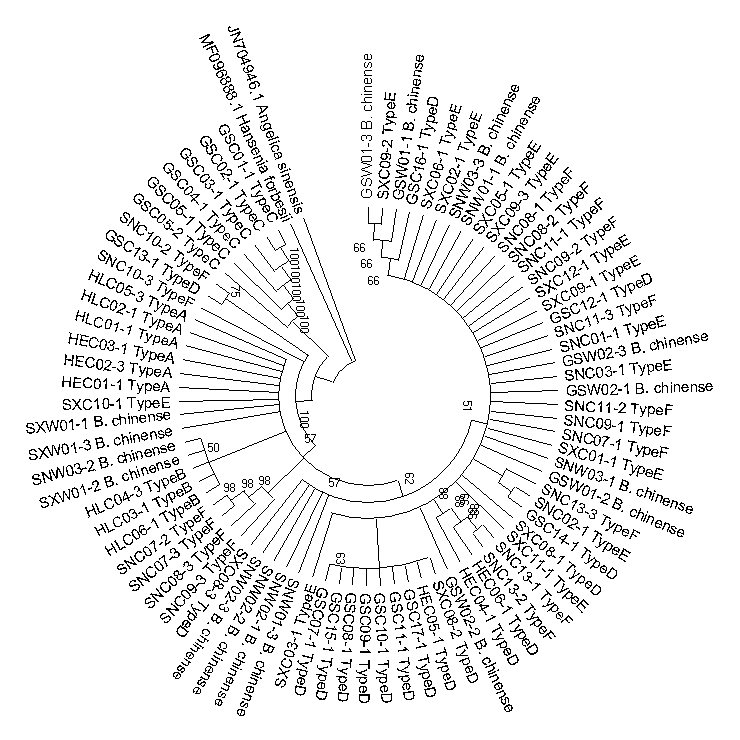

Supplement: Supplemental Information 1 — The bootstrap scores (1000 replicates) are shown for each branch. [file peerj-10-13208-s001.png]

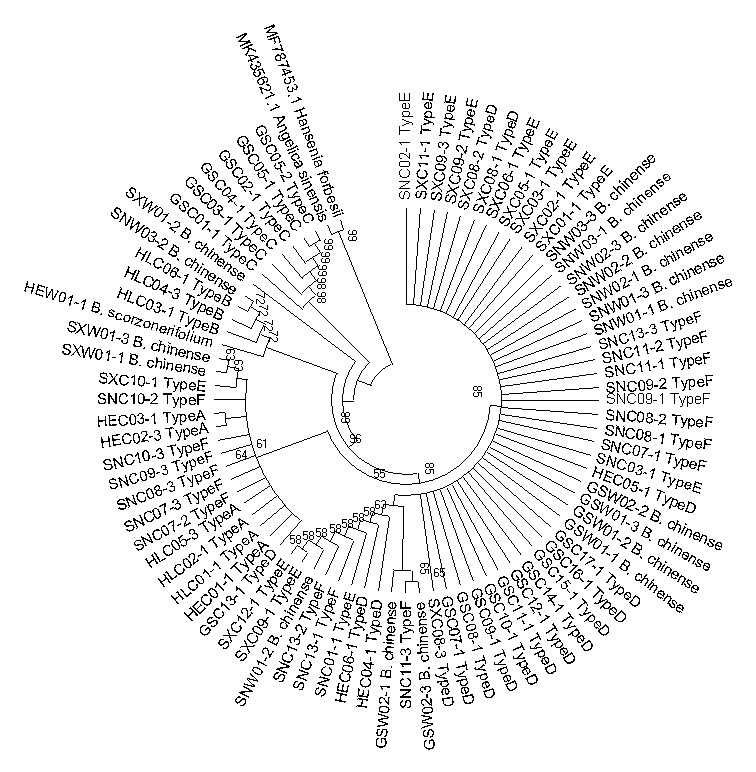

Supplement: Supplemental Information 2 — The bootstrap scores (1000 replicates) are shown for each branch. [file peerj-10-13208-s002.png]

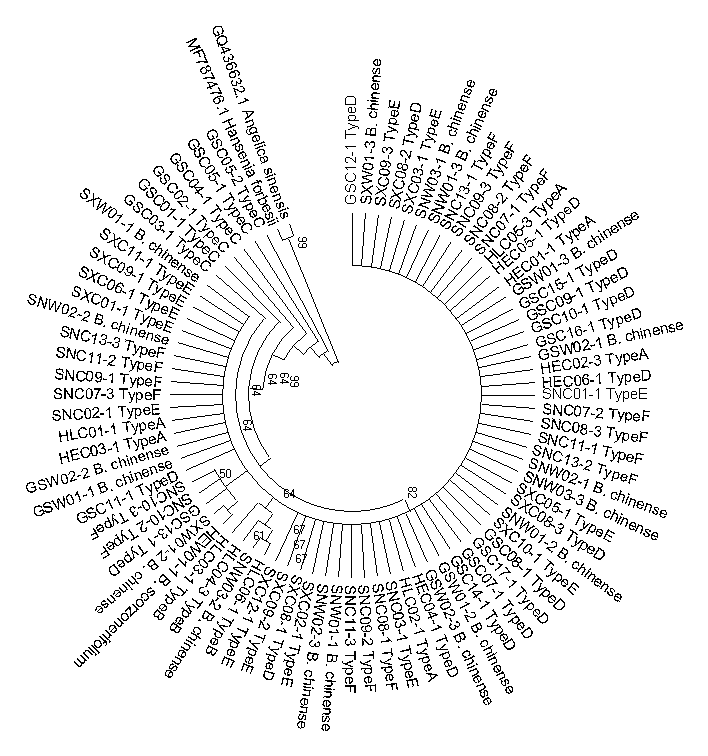

Supplement: Supplemental Information 3 — The bootstrap scores (1000 replicates) are shown for each branch. [file peerj-10-13208-s003.png]

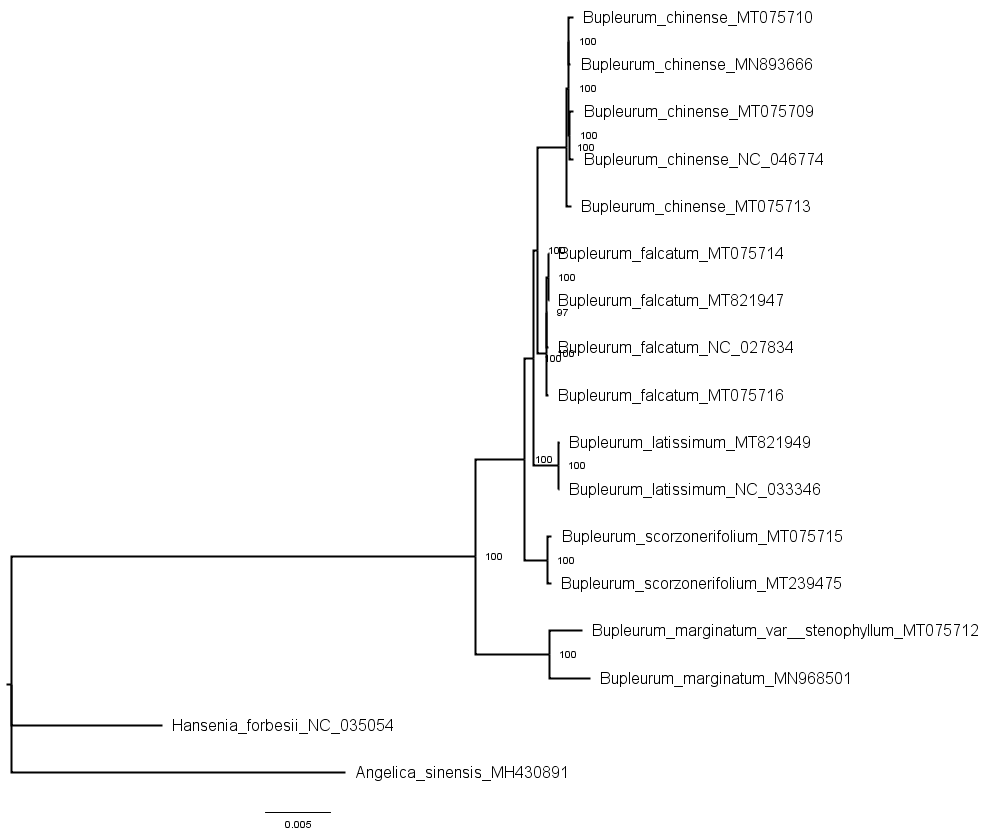

Supplement: Supplemental Information 4 [file peerj-10-13208-s004.png]

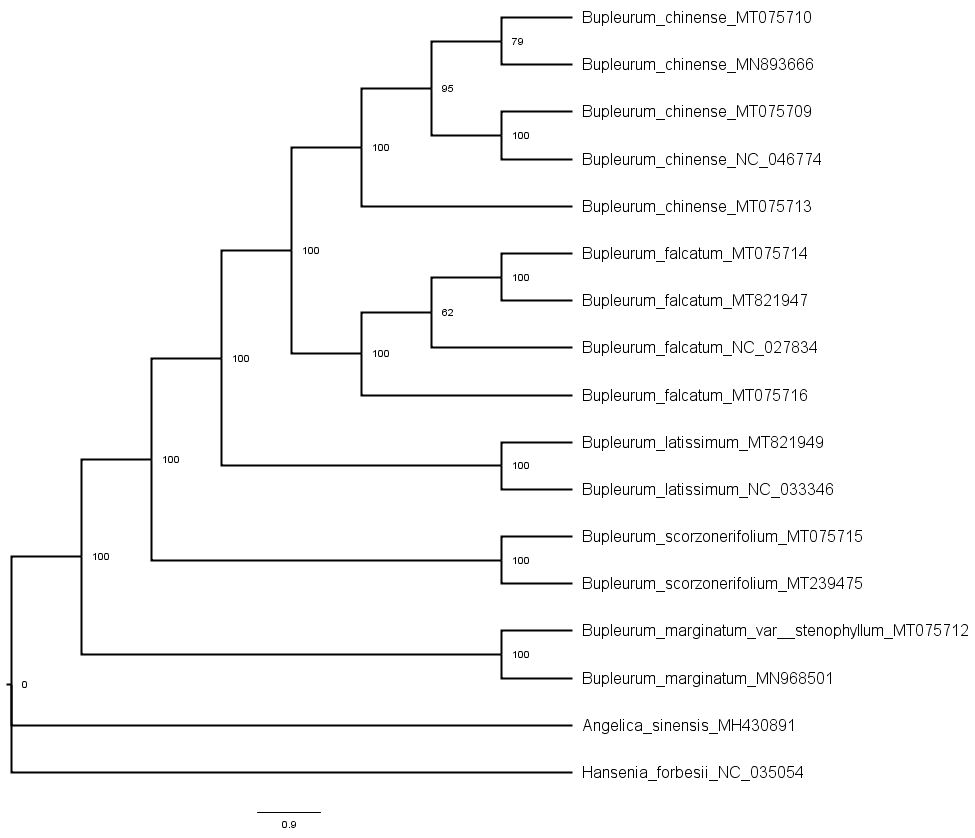

Supplement: Supplemental Information 5 [file peerj-10-13208-s005.png]

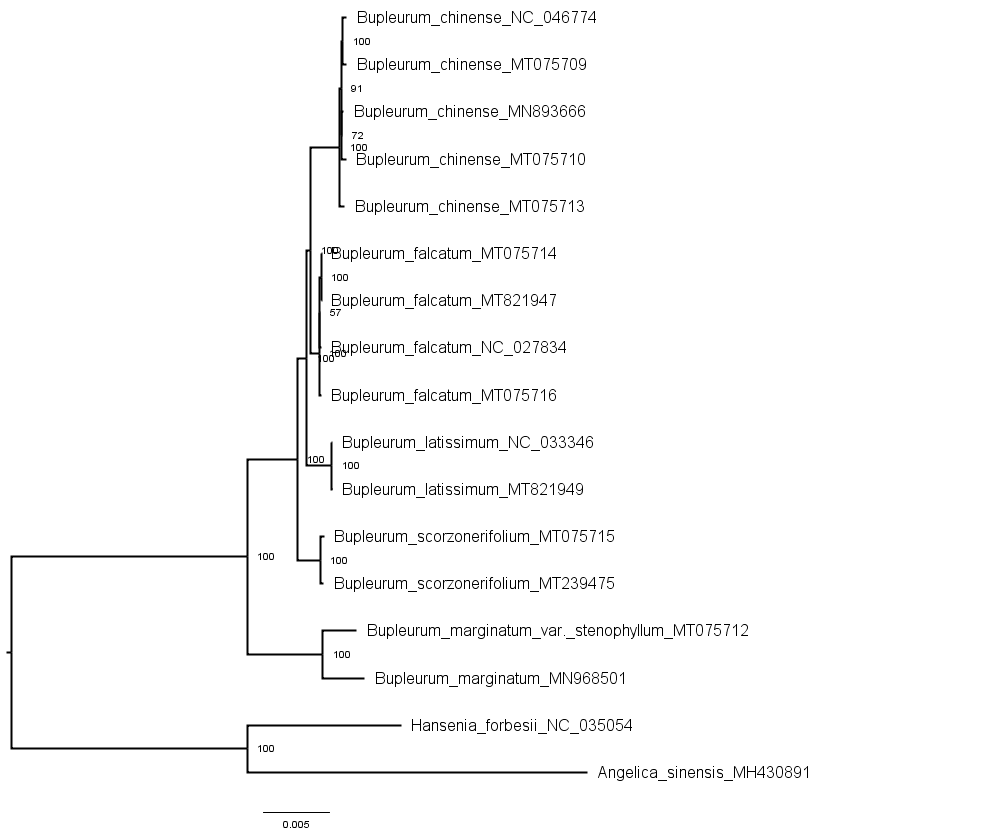

Supplement: Supplemental Information 6 [file peerj-10-13208-s006.png]

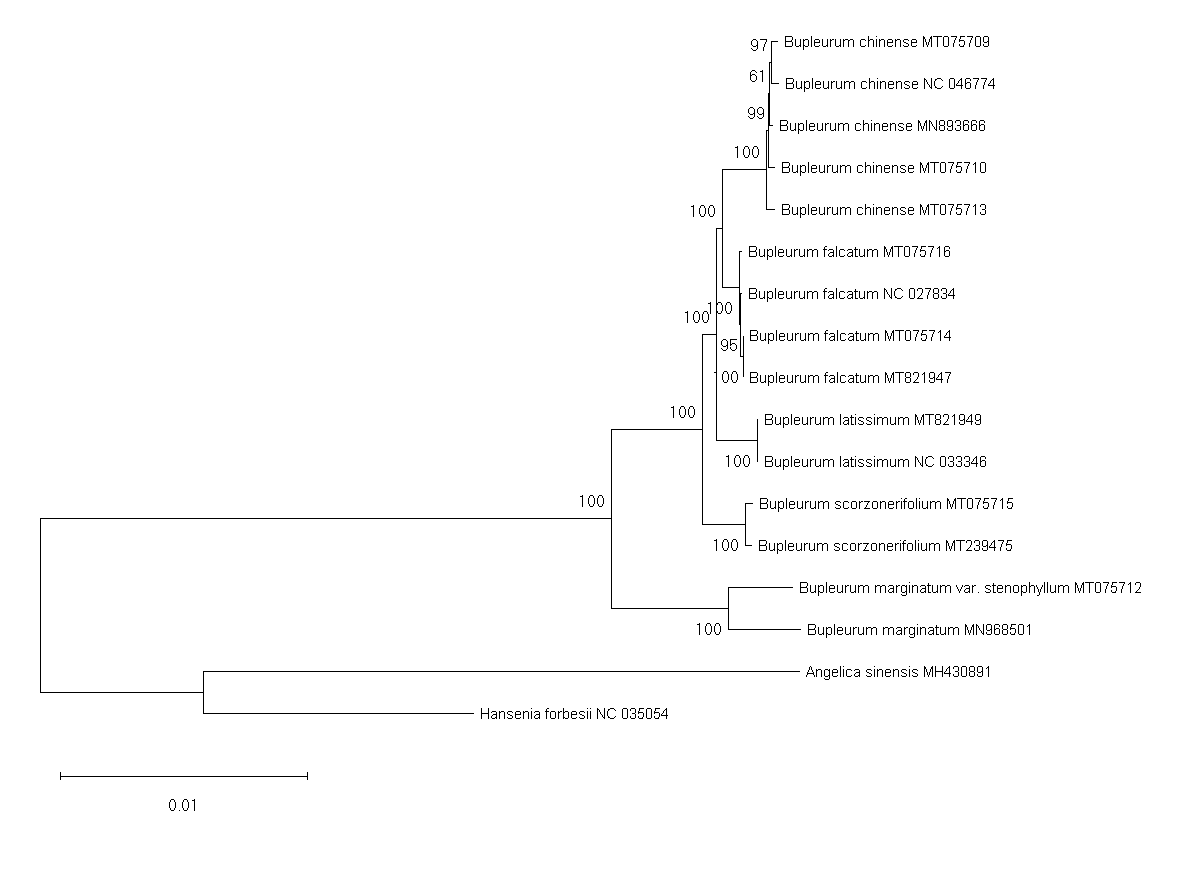

Supplement: Supplemental Information 7 [file peerj-10-13208-s007.png]

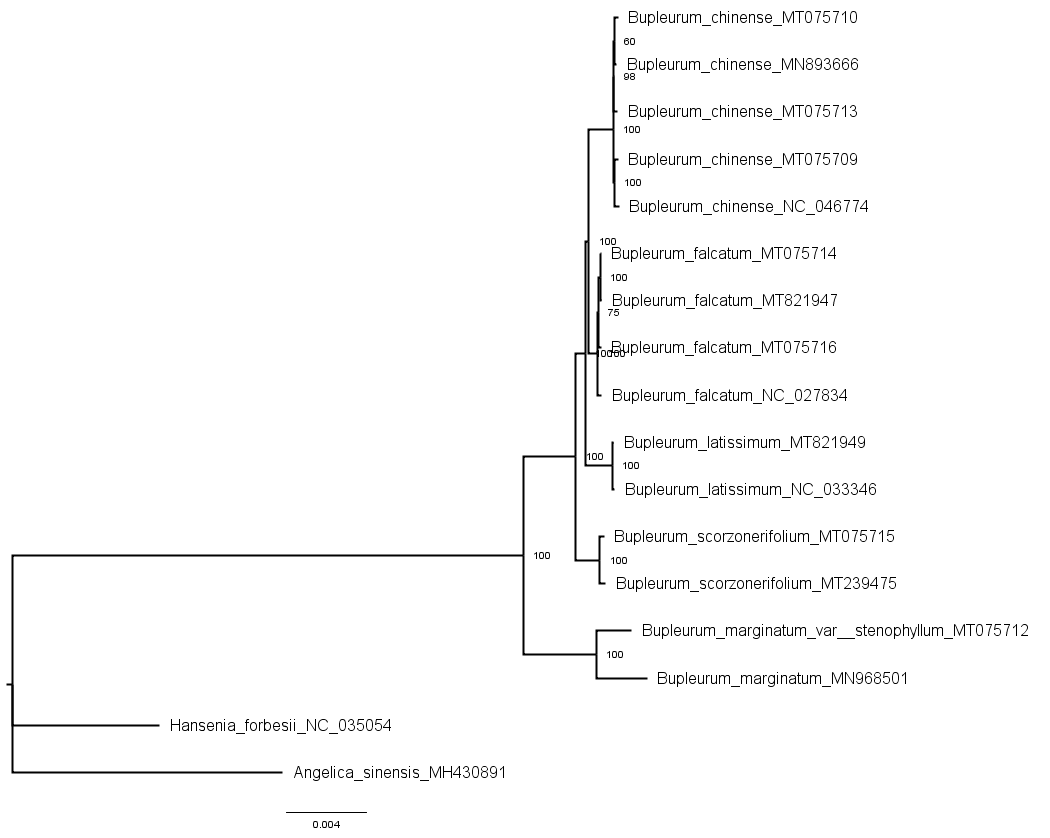

Supplement: Supplemental Information 8 [file peerj-10-13208-s008.png]

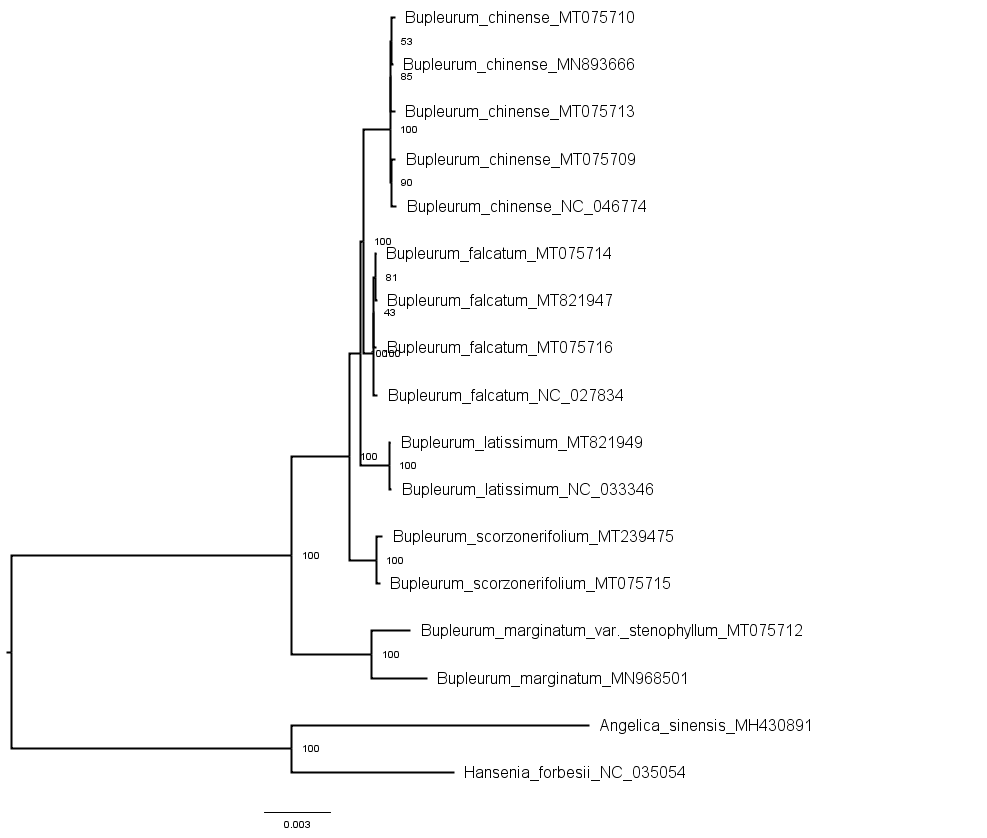

Supplement: Supplemental Information 9 [file peerj-10-13208-s009.png]

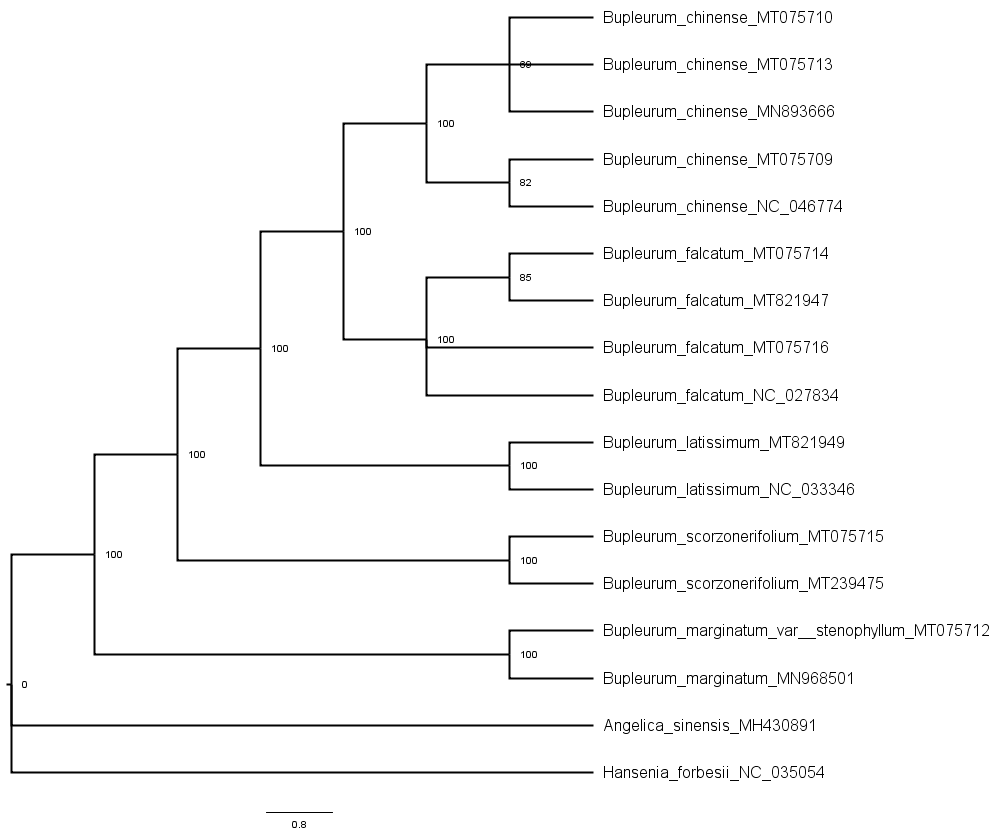

Supplement: Supplemental Information 10 [file peerj-10-13208-s010.png]

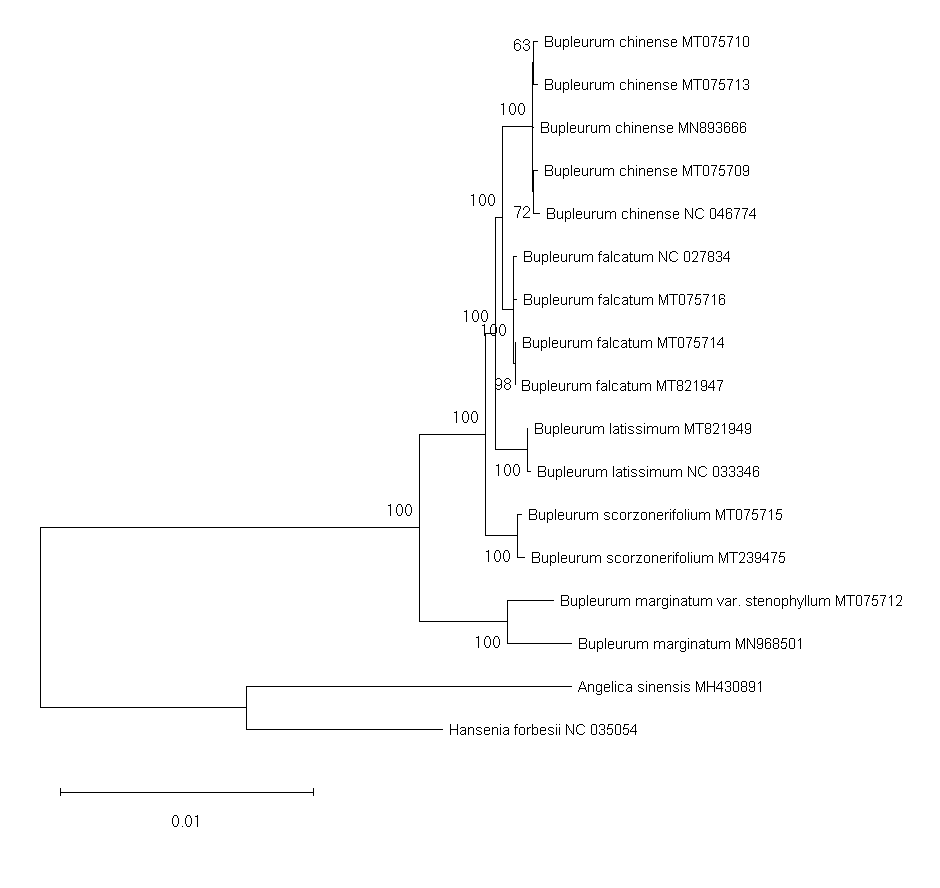

Supplement: Supplemental Information 11 [file peerj-10-13208-s011.png]
