# Supplementary material for: Identification of the original plants of cultivated Bupleuri Radix based on DNA barcoding and chloroplast genome analysis
Source: PeerJ. 2022 Apr 12;10:e13208. doi: 10.7717/peerj.13208 (PMC9012172; doi:10.7717/peerj.13208)
Supplement: Supplemental Information 12 [file peerj-10-13208-s012.docx]

| **No.** | **Location** | **Longitude and latitude** | **Serial population** | **Voucher** | **Sample code** |
| --- | --- | --- | --- | --- | --- |
| 1 | Yaxia Town, Lintao County, Dingxi City, Gansu Province | N35°12'12.7" E103°49'11.06" | GSC01 | G.X. Zhang 62000101~03 | GSC01-1~01-3 |
| 2 | Yaxia Town, Lintao County, Dingxi City, Gansu Province | N35°15'30.51" E103°48'40.20" | GSC02 | G.X. Zhang 62000201~03 | GSC02-1~02-3 |
| 3 | Yaxia Town, Lintao County, Dingxi City, Gansu Province | N35°14′5.67″ E103°44′1.44″ | GSC03 | G.X. Zhang 62000301~03 | GSC03-1~03-3 |
| 4 | Shouyang Town, Longxi County, Dingxi City, Gansu Province | N35°05′10.87″ E104°24′16.51″ | GSC04 | G.X. Zhang 62000401~03 | GSC04-1~04-3 |
| 5 | Xiacheng Town, Weiyuan County, Dingxi City, Gansu Province | N35°1'22.76" E103°51'38.86" | GSC05 | G.X. Zhang 62000501~03 | GSC05-1~05-3 |
| 6 | Mahe Town, Longxi County, Dingxi City, Gansu Province | N35°21'46.22" E104°37'23.97" | GSC06 | G.X. Zhang 62000601~03 | GSC06-1~06-3 |
| 7 | Shouyang Town, Longxi County, Dingxi City, Gansu Province | N35°05′19.27″ E104°24′50.12″ | GSC07 | G.X. Zhang 62000701~03 | GSC07-1~07-3 |
| 8 | Tong'anyi Town, Longxi County, Dingxi City, Gansu Province | N35°18'18.38" E104°41'16.67" | GSC08 | G.X. Zhang 62000801~03 | GSC08-1~08-3 |
| 9 | Lianlu Town, Kangle County, Linxia Hui Autonomous Prefecture, Gansu Province | N35°1′25.64" E103°45′47.94" | GSC09 | Zhang&Wang 62000901~03 | GSC09-1~09-3 |
| 10 | Lianlu Town, Kangle County, Linxia Hui Autonomous Prefecture, Gansu Province | N35°0′26.45" E103°46′40.24" | GSC10 | Zhang&Wang 62001001~03 | GSC10-1~10-3 |
| 11 | Lianlu Town, Kangle County, Linxia Hui Autonomous Prefecture, Gansu Province | N35°0′8.65" E103°46′52.96" | GSC11 | Zhang&Wang 62001101~03 | GSC11-1~11-3 |
| 12 | Sancha Town, Zhang County, Dingxi City, Gansu Province | N34°52'14.47" E104°19'35.28" | GSC12 | Zhang&Wang 62001201~03 | GSC12-1~12-3 |
| 13 | Sancha Town, Zhang County, Dingxi City, Gansu Province | N34°52'10.73" E104°19'27.69" | GSC13 | Zhang&Wang 62001301~03 | GSC13-1~13-3 |
| 14 | Yihuqiao Town, Zhang County, Dingxi City, Gansu Province | N34°52'0.43" E104°15'37.57" | GSC14 | Zhang&Wang 62001401~03 | GSC14-1~14-3 |
| 15 | Yangyong Town, Lintan County, Gannan Prefecture, Gansu Province | N34°39′33.54" E103°27′40.84" | GSC15 | Zhang&Wang 62001501~03 | GSC15-1~15-3 |
| 16 | Liulin Town, Zhuoni County, Gannan Prefecture, Gansu Province | N34°38′9.07" E103°29′38.42" | GSC16 | Zhang&Wang 62001601~03 | GSC16-1~16-3 |
| 17 | Liulin Town, Zhuoni County, Gannan Prefecture, Gansu Province | N34°38′6.41" E103°30′7.8" | GSC17 | Zhang&Wang 62001701~03 | GSC17-1~17-3 |
| 18 | Dayang Town, Zhangjiachuan County, Tianshui City, Gansu Province | N34°58′32.07″ E106°02′55.04″ | GSC18 | Zhang&Li 62001801~03 | GSC18-1~18-3 |
| 19 | Beiduancun Town, Anguo City, Baoding City, Hebei Province | N38°27′47.58″ E115°18′12.03″ | HEC01 | Zhang&Wang13000101~03 | HEC01-1~01-3 |
| 20 | Boye Town, Boye County, Baoding City, Hebei Province | N38°27′25.02″ E115°25′17.83″ | HEC02 | Zhang&Wang13000201~03 | HEC02-1~02-3 |
| 21 | Boye Town, Boye County, Baoding City, Hebei Province | N38°27′50.33″ E115°25′8.77″ | HEC03 | Zhang&Wang13000301~03 | HEC03-1~03-3 |
| 22 | Gengle Town, She County, Handan City, Hebei Province | N36°32′30.28″ E113°48′57.67″ | HEC04 | Zhang&Wang13000401~03 | HEC04-1~04-3 |
| 23 | Guxin Town, She County, Handan City, Hebei Province | N36°27'12.23" E113°41'33.29" | HEC05 | Zhang&Wang 13000501~03 | HEC05-1~05-3 |
| 24 | Piancheng Town, She County, Handan City, Hebei Province | N36°46′23.81" E113°41′15.99" | HEC06 | Zhang&Wang13000601~03 | HEC06-1~06-3 |
| 25 | Huayuan Town, Lindian County, Daqing City, Heilongjiang Province | N46°53′44.49" E124°55′43.07" | HLC01 | Zhang&Wang 23000101~03 | HLC01-1~01-3 |
| 26 | Huayuan Town, Lindian County, Daqing City, Heilongjiang Province | N46°54′13.13" E124°54′43.27" | HLC02 | Zhang&Wang 23000201~03 | HLC02-1~02-3 |
| 27 | Huayuan Town, Lindian County, Daqing City, Heilongjiang Province | N46°53′44.49" E124°55′43.07" | HLC03 | Zhang&Wang 23000301~03 | HLC03-1~03-3 |
| 28 | Huayuan Town, Lindian County, Daqing City, Heilongjiang Province | N46°58′47.38" E124°54′22.25" | HLC04 | Zhang&Wang 23000401,02,03 | HLC04-1~04-3 |
| 29 | Taipingzhuang Town, Anda City, Suihua City, Heilongjiang Province | N47°01′43.96″ E125°18′56.68″ | HLC05 | Zhang&Wang 23000501~03 | HLC05-1~05-3 |
| 30 | Taipingzhuang Town, Anda City, Suihua City, Heilongjiang Province | N47°01′43.96″ E125°18′56.68″ | HLC06 | Zhang&Wang 23000601~03 | HLC06-1~06-3 |
| 31 | Jiedian Town, Wanrong County, Yuncheng City, Shanxi Province | N35°25'4.22" E110°53'0.08" | SXC01 | Zhang&Wang 14000101~03 | SXC01-1~01-3 |
| 32 | Xicun Town, Wanrong County, Yuncheng City, Shanxi Province | N35°25'39.08" E110°55'23.55" | SXC02 | Zhang&Wang 14000201~03 | SXC02-1~02-3 |
| 33 | Xicun Town, Wanrong County, Yuncheng City, Shanxi Province | N35°25'33.73" E110°57'41.03" | SXC03 | Zhang&Wang 14000301~03 | SXC03-1~03-3 |
| 34 | Qinghe Town, Jishan County, Yuncheng City, Shanxi Province | N35°29'50.43" E111°1'49.53" | SXC04 | Zhang&Wang 14000401~03 | SXC04-1~04-3 |
| 35 | Qinghe Town, Jishan County, Yuncheng City, Shanxi Province | N35°30'6.6" E111°1'24.5" | SXC05 | Zhang&Wang 14000501~03 | SXC05-1~05-3 |
| 36 | Taiyang Town, Jishan County, Yuncheng City, Shanxi Province | N35°27'6.13" E111°0'32.9" | SXC06 | Zhang&Wang 14000601~03 | SXC06-1~06-3 |
| 37 | Miaoqian Town, Xia County, Yuncheng City, Shanxi Province | N35°2'7.88" E111°12'34.26" | SXC07 | Zhang&Wang 14000701~03 | SXC07-1~07-3 |
| 38 | Miaoqian Town, Xia County, Yuncheng City, Shanxi Province | N35°02′9.01″ E111°12′25.21″ | SXC08 | Zhang&Wang 14000801~03 | SXC08-1~08-3 |
| 39 | Nandali Town, Xia County, Yuncheng City, Shanxi Province | N35°12'26.64" E111°19'35.63" | SXC09 | Zhang&Wang 14000901~03 | SXC09-1~09-3 |
| 40 | Yangwang Town, Xinjiang County, Yuncheng City, Shanxi Province | N35°29'29.2" E111°2'41.81" | SXC10 | Zhang&Wang 14001001~03 | SXC10-1~10-3 |
| 41 | Yangwang Town, Xinjiang County, Yuncheng City, Shanxi Province | N35°29'28.25" E111°3'0.62" | SXC11 | Zhang&Wang 14001101~03 | SXC11-1~11-3 |
| 42 | Yangwang Town, Xinjiang County, Yuncheng City, Shanxi Province | N35°30'11.05" E111°3'25.93" | SXC12 | Zhang&Wang 14001201~03 | SXC12-1~12-3 |
| 43 | Zhuangtou Town, Chengcheng County, Weinan City, Shaanxi Province | N35°10'0.74" E110°0'7.37" | SNC01 | Zhang&Wang 61000101~03 | SNC01-1~01-3 |
| 44 | Zhuangtou Town, Chengcheng County, Weinan City, Shaanxi Province | N35°10'31.95" E109°58'54.51" | SNC02 | Zhang&Wang 61000201~03 | SNC02-1~02-3 |
| 45 | Zhuangtou Town, Chengcheng County, Weinan City, Shaanxi Province | N35°9'34.54" E109°58'48.97" | SNC03 | Zhang&Wang 61000301~03 | SNC03-1~03-3 |
| 46 | Fang Town, Heyang County, Weinan City, Shaanxi Province | N35°10′39.86″ E110°14′42.24″ | SNC04 | Zhang&Wang 61000401~03 | SNC04-1~04-3 |
| 47 | Fang Town, Heyang County, Weinan City, Shaanxi Province | N35°10′24.94″ E110°15′10.05″ | SNC05 | Zhang&Wang 61000501~03 | SNC05-1~05-3 |
| 48 | Fang Town, Heyang County, Weinan City, Shaanxi Province | N35°10′6.69″ E110°14′32.04″ | SNC06 | Zhang&Wang 61000601~03 | SNC06-1~06-3 |
| 49 | Yecun Town, Shangzhou District, Shangluo City, Shaanxi Province | N33°43′56.31" E110°8′54.56" | SNC07 | Zhang&Wang 61000701~03 | SNC07-1~07-3 |
| 50 | Yecun Town, Shangzhou District, Shangluo City, Shaanxi Province | N33°45′32.2" E110°9′24.82" | SNC08 | Zhang&Wang 61000801~03 | SNC08-1~08-3 |
| 51 | Yecun Town, Shangzhou District, Shangluo City, Shaanxi Province | N33°45′48.84" E110°9′51.37" | SNC09 | Zhang&Wang 61000901~03 | SNC09-1~09-3 |
| 52 | Tuoshi Town, Chencang District, Baoji City, Shaanxi Province | N34°30'21.07" E106°35'18.65" | SNC10 | Zhang&Wang 61001001~03 | SNC10-1~10-3 |
| 53 | Xiangong Town, Chencang District, Baoji City, Shaanxi Province | N34°35′33.24″ E107°01′13.89″ | SNC11 | Zhang&Wang 61001101~03 | SNC11-1~11-3 |
| 54 | Chisha Town, Chencang District, Baoji City, Shaanxi Province | N34°30′28.11″ E106°39′6.22″ | SNC12 | Zhang&Wang 61001201~03 | SNC12-1~12-3 |
| 55 | Caijiapo Town, Qishan County, Baoji City, Shaanxi Province | N34°10'53.02" E107°39'0.25" | SNC13 | Zhang&Wang 61001301~03 | SNC13-1~13-3 |
| 56 | Yingge Town, Taibai County, Baoji City, Shaanxi Province | N34°4'37.47" E107°39'34.07" | SNC14 | Zhang&Wang 61001401~03 | SNC14-1~14-3 |
| 57 | Tongda Town, Mingshui County, Suihua City, Heilongjiang Province | N47°10′26.63″ E125°24′14.93″ | HLW01 | Zhang&Wang 23100101~03 | HLW01-1~01-3 |
| 58 | Huayuan Town, Lindian County, Daqing City, Heilongjiang Province | N46°56′28.87″ E124°42′57.15″ | HLW02 | Zhang&Wang 23100201~03 | HLW02-1~02-3 |
| 59 | Anjiang Town, Chengde County, Chengde City, Hebei Province | N40°44′29.65″ E117°37′44.05″ | HEW01 | Zhang&Wang13100101~03 | HEW01-1~01-3 |
| 60 | Huichuan Town, Weiyuan County, Dingxi City, Gansu Province | N35°6′31.66″，E103°57′16.38″ | GSW01 | Zhang&Wang 62100101~03 | GSW01-1~01-3 |
| 61 | Dongcha Town, Maiji District, Tianshui City, Gansu Province | N34°24′44.17″ E106°37′4.31″ | GSW02 | Zhang&Wang 62100201~03 | GSW02-1~02-3 |
| 62 | Moli Town, Jiangxian County, Yuncheng City, Shanxi Province | N35°28′32.68″ E111°46′47.8″ | SXW01 | Zhang&Wang 14100101~03 | SXW01-1~01-3 |
| 63 | Zhuangtou Town, Chengcheng County, Weinan City, Shaanxi Province | N35°10′30.88″ E110°0′36.01″ | SNW01 | Zhang&Wang 61100101~03 | SNW01-1~01-3 |
| 64 | Shahezi Town, Shangzhou District, Shangluo City, Shaanxi Province | N33°48′41.66″ E110°2′21.92″ | SNW02 | Zhang&Wang 61100201~05 | SNW02-1~02-3 |
| 65 | Jinhe Town, Jintai District, Baoji City, Shaanxi Province | N34°24′25.49″ E107°4′54.97″ | SNW03 | Zhang&Wang 61100301~03 | SNW03-1~03-3 |
| 66 | Sangri Town, Sangri County, Shannan City, Tibet Autonomous Region | N29°18′52.75″ E92°05′35.42″ | XZW01 | Y.D. Qi 54100101~03 | XZW01-1~01-3 |
