# Supplementary material for: Identification of the original plants of cultivated Bupleuri Radix based on DNA barcoding and chloroplast genome analysis
Source: PeerJ. 2022 Apr 12;10:e13208. doi: 10.7717/peerj.13208 (PMC9012172; doi:10.7717/peerj.13208)
Supplement: Supplemental Information 13 — - indicates that there is no corresponding experiment for the sample. [file peerj-10-13208-s013.docx]

| **Specimen No.** | **GenBank accession No.** | | | |
| --- | --- | --- | --- | --- |
|  | **ITS** | ***psbA-trnH*** | ***matK*** | ***rbcL*** |
| GSC01-1 | MT074485 | MT085920 | MT085926 | MT086041 |
| GSC02-1 | MT074486 | MT085921 | MT085927 | MT086049 |
| GSC03-1 | MT074487 | MT085922 | MT085928 | MT086050 |
| GSC04-1 | MT074488 | MT085923 | MT085929 | MT086019 |
| GSC04-2 | MT074489 | - | - | - |
| GSC04-3 | MT074490 | - | - | - |
| GSC05-1 | MT074491 | MT085924 | MT085930 | MT086095 |
| GSC05-2 | MT074492 | MT085925 | MT085931 | MT086105 |
| GSC05-3 | MT074493 | - | - | - |
| GSC06-1 | MT074494 | - | - | - |
| GSC07-1 | MT074495 | MT085907 | MT085933 | MT086028 |
| GSC08-1 | MT074496 | MT085908 | MT085934 | MT086104 |
| GSC09-1 | MT074497 | MT085909 | MT085935 | MT086035 |
| GSC10-1 | MT074498 | MT085905 | MT085936 | MT086108 |
| GSC11-1 | MT074499 | MT085906 | MT085937 | MT086036 |
| GSC12-1 | MT074500 | MT085847 | MT085938 | MT086066 |
| GSC13-1 | MT074501 | MT085840 | MT085939 | MT086100 |
| GSC14-1 | MT074502 | MT085848 | MT085940 | MT086033 |
| GSC15-1 | MT074503 | MT085910 | MT085941 | MT086051 |
| GSC16-1 | MT074504 | MT085843 | MT085942 | MT086052 |
| GSC17-1 | MT074505 | MT085911 | MT085943 | MT086089 |
| GSC18-1 | MT074506 | - | - | - |
| GSC18-2 | MT074507 | - | - | - |
| GSC18-3 | MT074508 | - | - | - |
| GSW01-1 | MT074509 | MT085844 | MT085944 | MT086106 |
| GSW01-2 | MT074510 | MT085850 | MT085945 | MT086109 |
| GSW01-3 | MT074511 | MT085845 | MT085946 | MT086102 |
| GSW02-1 | MT074512 | MT085851 | MT085947 | MT086098 |
| GSW02-2 | MT074513 | MT085852 | MT085948 | MT086103 |
| GSW02-3 | MT074514 | MT085853 | MT085949 | MT086038 |
| HEC01-1 | MT074515 | MT085893 | MT085950 | MT086053 |
| HEC01-2 | MT074516 | - | - | - |
| HEC01-3 | MT074517 | - | - | - |
| HEC02-1 | MT074518 | - | - | - |
| HEC02-2 | MT074519 | - | - | - |
| HEC02-3 | MT074520 | MT085894 | MT085951 | MT086090 |
| HEC03-1 | MT074521 | MT085895 | MT085952 | MT086039 |
| HEC03-2 | MT074522 | - | - | - |
| HEC03-3 | MT074523 |  | - | - |
| HEC04-1 | MT074524 | MT085914 | MT085953 | MT086079 |
| HEC05-1 | MT074525 | MT085912 | MT085954 | MT086080 |
| HEC06-1 | MT074526 | MT085915 | MT085955 | MT086091 |
| HEW01-1 | MT074527 | - | MT085956 | MT086096 |
| HEW01-2 | MT074528 | - | - | - |
| HEW01-3 | MT074529 | - | - | - |
| HLC01-1 | MT074530 | MT085896 | MT085957 | MT086029 |
| HLC01-2 | MT074531 | - | - | - |
| HLC01-3 | MT074532 | - | - | - |
| HLC02-1 | MT074533 | MT085897 | MT085958 | MT086023 |
| HLC02-2 | MT074534 | - | - | - |
| HLC02-3 | MT074535 | - | - | - |
| HLC03-1 | MT074536 | MT085889 | MT085959 | MT086030 |
| HLC03-2 | MT074537 | - | - | - |
| HLC03-3 | MT074538 | - | - | - |
| HLC04-1 | MT074539 | - | - | - |
| HLC04-2 | MT074540 | - | - | - |
| HLC04-3 | MT074541 | MT085890 | MT085960 | MT086020 |
| HLC05-1 | MT074542 | - | - | - |
| HLC05-2 | MT074543 | - | - | - |
| HLC05-3 | MT074544 | MT085898 | MT085961 | MT086025 |
| HLC06-1 | MT074545 | MT085891 | MT085962 | MT086018 |
| HLC06-2 | MT074546 | - | - | - |
| HLC06-3 | MT074547 | - | - | - |
| HLW01-1 | MT074548 | - | - | - |
| HLW01-2 | MT074549 | - | - | - |
| HLW01-3 | MT074550 | - | - | - |
| HLW02-1 | MT074551 | - | - | - |
| HLW02-2 | MT074552 | - | - | - |
| HLW02-3 | MT074553 | - | - | - |
| SNC01-1 | MT074554 | MT085854 | MT085963 | MT086031 |
| SNC02-1 | MT074555 | MT085855 | MT085964 | MT086101 |
| SNC03-1 | MT074556 | MT085856 | MT085965 | MT086034 |
| SNC04-1 | MT074557 | - | - | - |
| SNC05-1 | MT074558 | - | - | - |
| SNC06-1 | MT074559 | - | - | - |
| SNC07-1 | MT074560 | MT085857 | MT085966 | MT086054 |
| SNC07-2 | MT074561 | MT085836 | MT085967 | MT086092 |
| SNC07-3 | MT074562 | MT085837 | MT085968 | MT086110 |
| SNC08-1 | MT074563 | MT085858 | MT085969 | MT086067 |
| SNC08-2 | MT074564 | MT085859 | MT085970 | MT086055 |
| SNC08-3 | MT074565 | MT085839 | MT085971 | MT086056 |
| SNC09-1 | MT074566 | MT085860 | MT085972 | MT086042 |
| SNC09-2 | MT074567 | MT085861 | MT085973 | MT086032 |
| SNC09-3 | MT074568 | MT085838 | MT085974 | MT086099 |
| SNC10-1 | MT074569 | - | - | - |
| SNC10-2 | MT074570 | MT085841 | MT085976 | MT086081 |
| SNC10-3 | MT074571 | MT085842 | MT085977 | MT086058 |
| SNC11-1 | MT074572 | MT085862 | MT085978 | MT086043 |
| SNC11-2 | MT074573 | MT085863 | MT085979 | MT086068 |
| SNC11-3 | MT074574 | MT085864 | MT085980 | MT086044 |
| SNC12-1 | MT074575 | - | - | - |
| SNC12-2 | MT074576 | - | - | - |
| SNC12-3 | MT074577 | - | - | - |
| SNC13-1 | MT074578 | MT085916 | MT085981 | MT086069 |
| SNC13-2 | MT074579 | MT085917 | MT085982 | MT086070 |
| SNC13-3 | MT074580 | MT085865 | MT085983 | MT086107 |
| SNC14-1 | MT074581 | - | - | - |
| SNC14-2 | MT074582 | - | - | - |
| SNC14-3 | MT074583 | - | - | - |
| SNW01-1 | MT074584 | MT085866 | MT085984 | MT086059 |
| SNW01-2 | MT074585 | - | MT085985 | MT086037 |
| SNW01-3 | MT074586 | MT085883 | MT085986 | MT086060 |
| SNW02-1 | MT074587 | MT085884 | MT085987 | MT086071 |
| SNW02-2 | MT074588 | MT085885 | MT085988 | MT086045 |
| SNW02-3 | MT074589 | MT085886 | MT085989 | MT086046 |
| SNW03-1 | MT074590 | MT085869 | MT085991 | MT086061 |
| SNW03-2 | MT074591 | MT085892 | MT085992 | MT086047 |
| SNW03-3 | MT074592 | MT085870 | MT085993 | MT086074 |
| SXC01-1 | MT074593 | MT085871 | MT085994 | MT086062 |
| SXC02-1 | MT074594 | MT085872 | MT085995 | MT086063 |
| SXC03-1 | MT074595 | MT085873 | MT085996 | MT086075 |
| SXC04-1 | MT074596 | - | - | - |
| SXC05-1 | MT074597 | MT085874 | MT085998 | MT086093 |
| SXC06-1 | MT074598 | MT085875 | MT085999 | MT086027 |
| SXC08-1 | MT074599 | MT085876 | MT086000 | MT086076 |
| SXC08-2 | MT074600 | MT085913 | MT086001 | MT086082 |
| SXC08-3 | MT074601 | MT085888 | MT086002 | MT086083 |
| SXC09-1 | MT074602 | MT085877 | MT086003 | MT086084 |
| SXC09-2 | MT074603 | MT085846 | MT086004 | MT086026 |
| SXC09-3 | MT074604 | MT085878 | MT086005 | MT086021 |
| SXC10-1 | MT074605 | MT085899 | MT086006 | MT086048 |
| SXC11-1 | MT074606 | MT085879 | MT086007 | MT086065 |
| SXC12-1 | MT074607 | MT085880 | MT086008 | MT086040 |
| SXW01-1 | MT074608 | MT085900 | MT086009 | MT086094 |
| SXW01-2 | MT074609 | MT085902 | MT086010 | MT086085 |
| SXW01-3 | MT074610 | MT085901 | MT086011 | MT086077 |
| XZW01-1 | MT074614 | - | - | - |
| XZW01-2 | MT074615 | - | - | - |
| XZW01-3 | MT074616 | - | - | - |
