# Supplementary material for: Identification of the original plants of cultivated Bupleuri Radix based on DNA barcoding and chloroplast genome analysis
Source: PeerJ. 2022 Apr 12;10:e13208. doi: 10.7717/peerj.13208 (PMC9012172; doi:10.7717/peerj.13208)
Supplement: Supplemental Information 14 [file peerj-10-13208-s014.docx]

| **No.** | **Code** | **Species** | **Location** |
| --- | --- | --- | --- |
| 1 | HEC02-3 | *B.falcatum* | Boye,Hebei,China |
| 2 | HEC01-1 | *B.falcatum* | Boye,Hebei,China |
| 3 | HEC03-1 | *B.falcatum* | Boye,Hebei,China |
| 4 | HLC01-1 | *B.falcatum* | Lindian,Heilongjiang,China |
| 5 | HLC02-2 | *B.falcatum* | Lindian,Heilongjiang,China |
| 6 | HLC05-3 | *B.falcatum* | Mingshui,Heilongjiang,China |
| 7 | HLC03-1 | *B.scorzonerifolium* | Mingshui,Heilongjiang,China |
| 8 | HLC06-2 | *B.scorzonerifolium* | Mingshui,Heilongjiang,China |
| 9 | HLC04-3 | *B.scorzonerifolium* | Lindian,Heilongjiang,China |
| 10 | GSC03-2 | *B.marginatum* var.*stenophyllum* | Lintao,Gansu,China |
| 11 | GSC04-2 | *B.marginatum* var.*stenophyllum* | Longxi,Gansu,China |
| 12 | GSC05-1 | *B.marginatum* var.*stenophyllum* | Weiyuan,Gansu,China |
| 13 | GSC12-1 | *B.chinense* | Zhangxia,Gansu,China |
| 14 | GSC06-2 | *B.chinense* | Longxi,Gansu,China |
| 15 | GSC18-3 | *B.chinense* | Qinan, Gansu,China |
| 16 | SXC12-1 | *B.chinense* | Xinjiang, Shanxi,China |
| 17 | SXC09-2 | *B.chinense* | Xiaxian,Shanxi,China |
| 18 | SXC06-1 | *B.chinense* | Jishan, Shanxi,China |
| 19 | SNC14-3 | *B.chinense* | Taibai, Shaanxi,China |
| 20 | SNC10-1 | *B.chinense* | Chencang, Shaanxi,China |
| 21 | SNC11-3 | *B.chinense* | Chencang, Shaanxi,China |
