# Supplementary material for: Identification of the original plants of cultivated Bupleuri Radix based on DNA barcoding and chloroplast genome analysis
Source: PeerJ. 2022 Apr 12;10:e13208. doi: 10.7717/peerj.13208 (PMC9012172; doi:10.7717/peerj.13208)
Supplement: Supplemental Information 15 [file peerj-10-13208-s015.docx]

| **No.** | **Gene** | **Primers** | **Sequence** |
| --- | --- | --- | --- |
| 1 | *rpl36_infA* | R-r36_iA | 5' TGTTCCTCCTCCATCAGTT 3' |
|  |  | F-r36_iA | 5' TCCCTCTTCTTGTACCCTT 3' |
| 2 | *rpoA_rps11* | R-rA_r11 | 5' GCTCAAACCGCAGCAGGA 3' |
|  |  | F-rA_r11 | 5' TTCGCATCGCAATGCCTAT 3' |
| 3 | *petD-CDS2_rpoA* | R-pDC2 | 5' CAATCAGAACTACCTCCCAGA 3' |
|  |  | F-pDC2 | 5' GAATGGTATTTCTTTCCCGTAT 3' |
| 4 | *psbH_petB-CDS1* | R-pHpB | 5' ATCATCACTTATTCCACGA 3' |
|  |  | F-pHpB | 5' ATGGTAAAGTAGCTCCTGG 3' |
| 5 | *psbB_psbT* | R-pBpT | 5' AGTCGAGACTAAGAGGAATGTA 3' |
|  |  | F-pBpT | 5' AGATTTGGATGCTCAAGTAGAA 3' |
| 6 | *rps18_rpl20* | R-r18_r20 | 5' TCGTCTCATCGGGATAG 3' |
|  |  | F-r18_r20 | 5' GATTTATTAGCGAACAAGG 3' |
| 7 | *psaJ_rpl33* | R-pJ_r33 | 5' TAACCGTTATTCGGACAT 3' |
|  |  | F-pJ_r33 | 5' TCTATGGTTCGGGTCTTT 3' |
| 8 | *trnP-UGG_psaJ* | R-tppj | 5' ATGTCAACGCATCTGGGAAA 3' |
|  |  | F-tppj | 5' AGGACGAAATCACGCTCTGT 3' |
| 9 | *petA_psbJ* | R-pA_Pj | 5' AAATGGCTGATACTACTGG 3' |
|  |  | F-pA_Pj | 5' TCTTCTTGGCATCTGTTA 3' |
| 10 | *ycf4_cemA* | R-y4_Ca | 5' ATTGGAGCGTTTCTTTGTGG 3' |
|  |  | F-y4_cA | 5' CATTCCCTTGACTCGTACTGA 3' |
| 11 | *psaI_ycf4* | R-pI_y4 | 5' ACCATTCCTTCTTTTCTATC 3' |
|  |  | F-pI_y4 | 5' TGTTTTGGTCCCTTTAGTAG 3' |
| 12 | *trnV-UAC-CDS1_trnM-CAU* | R-tV-tM | 5' TCACAAGAAGCCCAGCAA 3' |
|  |  | F-tV-tM | 5' CGCGTGTAAACGAGGTGC 3' |
| 13 | *ndhC_trnV-UAC-CDS2* | R-nC_tV | 5' GACCTACTTCAAGATAACCC 3' |
|  |  | F-nC_tV | 5' CACCTAATACATCGAAACTC 3' |
| 14 | *trnD-GUC_trnY-GUA* | R-tD_tY | 5' ACAAAGAGGTTCTTCTTGGGT 3' |
|  |  | F-tD_tY | 5' ACTTATTGAATCTGTCGGGACT 3' |
| 15 | *ndhG_ndhI* | R-nG_nI | 5' GGAAATTGGAAAACGATA 3' |
|  |  | F-nG_nI | 5' ATACCACTCCCAGACCTC 3' |
| 16 | *ndhE_ndhG* | R-nE_nG | 5' GGGTTACATCGGTGGTT 3' |
|  |  | F-nE_nG | 5' CTTCAGCGGCTGCAATA 3' |
| 17 | *ndhD_psaC* | R-nD_pC | 5' TTCTTGAGTGTTCGGGTTTCT 3' |
|  |  | F-nD_pC | 5' TAATCCTATTTGCCCGCTGTA 3' |
| 18 | *rpl32_trnL-UAG* | R-r32_tL | 5' CGGACTCGAACCGAGATGCTA 3' |
|  |  | F-r32_tL | 5' TTGGAAGGGGAAGGGGTATTG 3' |
| 19 | *ndhF_rpl32* | R-nF_r32 | 5' TAAGGCTGCCCAATAC 3' |
|  |  | F-nF_r32 | 5' CGATCAAGTAACCGAATT 3' |
