# Supplementary material for: Identification of the original plants of cultivated Bupleuri Radix based on DNA barcoding and chloroplast genome analysis
Source: PeerJ. 2022 Apr 12;10:e13208. doi: 10.7717/peerj.13208 (PMC9012172; doi:10.7717/peerj.13208)
Supplement: Supplemental Information 16 [file peerj-10-13208-s016.docx]

| Type | 0 | 0 | 0 | 0 | 0 | 1 | 1 | 1 | 1 | 2 |  | 2 | 2 | 2 | 2 | 2 | 3 | 3 | 3 | 3 | 3 |  | 3 | 3 | 3 | 3 | 3 | 3 | 4 | 4 | No. |
| --- | --- | --- | --- | --- | --- | --- | --- | --- | --- | --- | --- | --- | --- | --- | --- | --- | --- | --- | --- | --- | --- | --- | --- | --- | --- | --- | --- | --- | --- | --- | --- |
|  | 2 | 5 | 5 | 5 | 5 | 2 | 6 | 8 | 9 | 5 |  | 5 | 6 | 8 | 9 | 9 | 1 | 1 | 1 | 1 | 2 |  | 3 | 4 | 5 | 6 | 9 | 9 | 1 | 1 |  |
|  | 7 | 0 | 1 | 3 | 4 | 3 | 5 | 7 | 6 | 1 |  | 3 | 5 | 0 | 2 | 8 | 3 | 4 | 5 | 9 | 8 |  | 6 | 9 | 6 | 9 | 5 | 7 | 4 | 6 |  |
| *B. chinense* | C | T | A | A | A | T | T | T | A | T |  | T | T | T | C | G | A | A | T | T | T |  | A | G | A | G | G | C | G | G | SNW01-1 |
| TypeE | **.** | **.** | **.** | **.** | **.** | **.** | **.** | **.** | **.** | **.** |  | **.** | **.** | **.** | **.** | **.** | **.** | **.** | **.** | **.** | **.** |  | **.** | **.** | **.** | **.** | **.** | **.** | **.** | **.** | SNC03-1、SXC09-3、SXC05-1、SXC01-1 |
| TypeD | **.** | **.** | **.** | **.** | **.** | **.** | **.** | - | **.** | **.** |  | **.** | **.** | **.** | **.** | **.** | **.** | **.** | **.** | **.** | **.** |  | - | - | **.** | **.** | **.** | **.** | **.** | **.** | SXC08-1 |
| TypeE | **.** | **.** | **.** | **.** | **.** | **.** | **.** | - | **.** | **.** |  | **.** | **.** | **.** | **.** | **.** | **.** | **.** | **.** | **.** | **.** |  | - | - | **.** | **.** | **.** | **.** | **.** | **.** | SXC11-1、SXC03-1 |
| *B****.*** *chinense* | **.** | **.** | **.** | **.** | **.** | **.** | **.** | - | **.** | **.** |  | **.** | **.** | **.** | **.** | **.** | **.** | **.** | **.** | **.** | **.** |  | - | - | **.** | **.** | **.** | **.** | **.** | **.** | GSW02-2 |
| TypeF | **.** | **.** | **.** | **.** | **.** | **.** | **.** | - | **.** | **.** |  | **.** | **.** | **.** | **.** | **.** | **.** | **.** | **.** | **.** | **.** |  | **.** | **.** | **.** | **.** | **.** | **.** | **.** | **.** | SNC11-3 |
| TypeE | **.** | **.** | **.** | **.** | **.** | **.** | **.** | - | **.** | **.** |  | **.** | **.** | **.** | **.** | **.** | **.** | **.** | **.** | **.** | **.** |  | **.** | **.** | **.** | **.** | **.** | **.** | **.** | **.** | SNC01-1、SXC12-1、SXC09-1、SXC02-1、SXC06-1 |
| *B. chinense* | **.** | **.** | **.** | **.** | **.** | **.** | **.** | - | **.** | **.** |  | **.** | **.** | **.** | **.** | **.** | **.** | **.** | **.** | **.** | **.** |  | **.** | **.** | **.** | **.** | **.** | **.** | **.** | **.** | GSW02-3、GSW02-1 |
| TypeD | **.** | **.** | **.** | **.** | **.** | **.** | **.** | - | **.** | **.** |  | **.** | **.** | **.** | **.** | **.** | **.** | **.** | **.** | **.** | **.** |  | **.** | **.** | **.** | **.** | **.** | **.** | **.** | **.** | GSC12-1 |
| TypeF | **.** | **.** | **.** | **.** | **.** | **.** | **.** | **.** | **.** | **.** |  | **.** | **.** | **.** | **.** | **.** | **.** | **.** | **.** | **.** | **.** |  | **.** | **.** | **.** | **.** | **.** | **.** | **.** | **.** | SNC11-2、SNC11-1、SNC09-2、SNC09-1、SNC08-2、SNC08-1、SNC07-1 |
| *B. chinense* | **.** | **.** | **.** | **.** | **.** | **.** | **.** | **.** | **.** | **.** |  | **.** | **.** | **.** | **.** | **.** | **.** | **.** | **.** | **.** | **.** |  | **.** | **.** | **.** | **.** | **.** | **.** | **.** | **.** | SNW03-1、SNW03-3、GSW01-2 |
| *B. chinense* | **.** | **.** | **.** | **.** | **.** | **.** | **.** | - | **.** | **.** |  | **.** | **.** | **.** | **.** | **.** | **.** | **.** | G | **.** | **.** |  | **.** | **.** | **.** | **.** | **.** | **.** | **.** | **.** | GSW01-1、GSW01-3 |
| TypeD | **.** | **.** | **.** | **.** | **.** | **.** | **.** | - | **.** | **.** |  | **.** | **.** | **.** | T | **.** | **.** | **.** | **.** | **.** | **.** |  | - | - | **.** | **.** | **.** | **.** | **.** | **.** | GSC15-1、SXC08-2、HEC05-1、GSC17-1、GSC11-1、GSC10-1、GSC09-1、GSC08-1、GSC07-1 |
| TypeF | **.** | **.** | **.** | **.** | **.** | **.** | **.** | - | T | **.** |  | **.** | **.** | **.** | **.** | **.** | **.** | **.** | **.** | **.** | **.** |  | - | - | **.** | **.** | **.** | **.** | **.** | **.** | SNC13-3 |
| TypeE | **.** | **.** | **.** | **.** | **.** | **.** | **.** | - | T | **.** |  | **.** | **.** | **.** | **.** | **.** | **.** | **.** | **.** | **.** | **.** |  | - | - | **.** | **.** | **.** | **.** | **.** | **.** | SNC02-1 |
| TypeD | **.** | **.** | **.** | **.** | **.** | **.** | **.** | - | T | **.** |  | **.** | **.** | **.** | **.** | **.** | **.** | **.** | **.** | **.** | **.** |  | - | - | **.** | **.** | **.** | **.** | **.** | **.** | GSC14-1 |
| TypeD | **.** | **.** | **.** | **.** | **.** | **.** | **.** | - | **.** | **.** |  | **.** | **.** | **.** | **.** | **.** | **.** | **.** | G | **.** | **.** |  | **.** | **.** | **.** | **.** | **.** | **.** | **.** | **.** | GSC16-1 |
| TypeE | **.** | **.** | **.** | **.** | **.** | **.** | **.** | - | **.** | **.** |  | **.** | **.** | **.** | **.** | **.** | **.** | **.** | G | **.** | **.** |  | **.** | **.** | **.** | **.** | **.** | **.** | **.** | **.** | SXC09-2 |
| *B. chinense* | **.** | **.** | **.** | **.** | **.** | **.** | **.** | - | **.** | **.** |  | **.** | **.** | **.** | **.** | **.** | **.** | **.** | **.** | **.** | **.** |  | T | **.** | **.** | **.** | **.** | **.** | **.** | **.** | SNW01-3、SNW02-1、SNW02-2、SNW02-3 |
| TypeD | **.** | **.** | **.** | **.** | **.** | **.** | **.** | - | **.** | **.** |  | **.** | **.** | **.** | **.** | **.** | **.** | **.** | **.** | **.** | **.** |  | T | **.** | **.** | **.** | **.** | **.** | **.** | **.** | SXC08-3 |
| TypeD | **.** | **.** | **.** | **.** | **.** | **.** | **.** | - | **.** | **.** |  | **.** | **.** | **.** | **.** | **.** | **.** | **.** | **.** | **.** | A |  | **.** | T | **.** | **.** | **.** | **.** | **.** | **.** | HEC04-1、HEC06-1 |
| TypeF | **.** | **.** | **.** | **.** | **.** | **.** | **.** | - | **.** | **.** |  | **.** | **.** | **.** | **.** | **.** | **.** | **.** | **.** | **.** | A |  | **.** | T | **.** | **.** | **.** | **.** | **.** | **.** | SNC13-1、SNC13-2 |
| TypeB | **.** | **.** | **.** | **.** | **.** | **.** | **.** | **.** | **.** | **.** |  | **.** | **.** | **.** | **.** | **.** | **.** | **.** | **.** | **.** | **.** |  | - | **.** | C | **.** | **.** | A | **.** | **.** | HLC06-1、HLC03-1 |
| TypeA | **.** | **.** | **.** | **.** | **.** | **.** | **.** | - | **.** | **.** |  | **.** | **.** | **.** | **.** | **.** | **.** | **.** | **.** | **.** | **.** |  | T | **.** | **.** | **.** | **.** | A | **.** | **.** | HEC01-1、HEC02-3、HEC03-1、HLC01-1、HLC02-1、HLC05-3 |
| *B. chinense* | **.** | **.** | **.** | **.** | **.** | **.** | **.** | **.** | **.** | **.** |  | **.** | **.** | **.** | **.** | **.** | **.** | **.** | **.** | **.** | **.** |  | T | **.** | **.** | **.** | **.** | A | **.** | **.** | SXW01-3、SXW01-1 |
| TypeE | **.** | **.** | **.** | **.** | **.** | **.** | **.** | **.** | **.** | **.** |  | **.** | **.** | **.** | **.** | **.** | **.** | **.** | **.** | **.** | **.** |  | T | **.** | **.** | **.** | **.** | A | **.** | **.** | SXC10-1 |
| *B****.*** *chinense* | **.** | **.** | **.** | **.** | **.** | **.** | **.** | - | **.** | **.** |  | **.** | **.** | **.** | **.** | **.** | **.** | **.** | **.** | **.** | **.** |  | T | **.** | C | **.** | **.** | A | **.** | **.** | SNW03-2 |
| TypeB | **.** | **.** | **.** | **.** | **.** | **.** | **.** | **.** | **.** | **.** |  | **.** | **.** | **.** | **.** | **.** | **.** | **.** | **.** | **.** | **.** |  | T | **.** | C | **.** | **.** | A | **.** | **.** | HLC04-3 |
| *B. chinense* | **.** | **.** | **.** | **.** | **.** | **.** | **.** | - | **.** | **.** |  | **.** | **.** | **.** | **.** | **.** | C | **.** | **.** | **.** | **.** |  | T | **.** | C | **.** | **.** | A | **.** | **.** | SXW01-2 |
| TypeF | **.** | **.** | **.** | **.** | **.** | **.** | **.** | - | **.** | **.** |  | **.** | **.** | **.** | **.** | T | **.** | **.** | **.** | A | **.** |  | T | **.** | **.** | **.** | **.** | A | **.** | **.** | SNC10-3 |
| TypeD | **.** | **.** | **.** | **.** | **.** | **.** | **.** | - | **.** | **.** |  | **.** | A | **.** | **.** | T | **.** | **.** | **.** | **.** | **.** |  | T | **.** | **.** | **.** | **.** | A | **.** | **.** | GSC13-1 |
| TypeF | **.** | **.** | **.** | **.** | **.** | **.** | **.** | - | **.** | **.** |  | **.** | A | **.** | **.** | T | **.** | **.** | **.** | **.** | **.** |  | T | **.** | **.** | **.** | **.** | A | **.** | **.** | SNC10-2 |
| TypeF | **.** | **.** | T | T | T | **.** | **.** | **.** | **.** | **.** |  | **.** | **.** | **.** | **.** | **.** | **.** | **.** | **.** | **.** | **.** |  | - | - | **.** | T | **.** | A | **.** | **.** | SNC09-3、SNC08-3、SNC07-3、SNC07-2 |
| TypeC | T | A | **.** | C | **.** | G | A | C | C | G |  | G | **.** | C | **.** | T | **.** | G | - | **.** | **.** |  | - | **.** | C | **.** | T | A | A | A | GSC05-2、GSC05-1、GSC04-1、GSC03-1、GSC02-1、GSC01-1 |
