# Supplementary material for: Identification of the original plants of cultivated Bupleuri Radix based on DNA barcoding and chloroplast genome analysis
Source: PeerJ. 2022 Apr 12;10:e13208. doi: 10.7717/peerj.13208 (PMC9012172; doi:10.7717/peerj.13208)
Supplement: Supplemental Information 18 [file peerj-10-13208-s018.docx]

| Type | 1 | 3 | 3 | 4 | 4 | 4 | No. |
| --- | --- | --- | --- | --- | --- | --- | --- |
|  | 6 | 7 | 9 | 0 | 7 | 8 |  |
|  | 6 | 0 | 2 | 4 | 5 | 4 |  |
| *B. chinense* | T | G | C | A | T | T | SNW01-1 |
| TypeD | **.** | **.** | **.** | **.** | **.** | **.** | GSC17-1、GSC07-1、GSC08-1、GSC09-1、GSC10-1、GSC11-1、GSC14-1、GSC15-1、GSC16-1、HEC04-1、HEC05-1、HEC06-1、SXC08-1、SXC08-2、SXC08-3 |
| *B. chinense* | **.** | **.** | **.** | **.** | **.** | **.** | GSW01-1、GSW01-2、GSW01-3、GSW02-1、GSW02-2、GSW02-3、SNW01-3、SNW02-1、SNW02-2、SNW02-3、SNW03-1、SNW03-3、SXW01-1、SXW01-3 |
| TypeA | **.** | **.** | **.** | **.** | **.** | **.** | HEC01-1、HEC02-3、HEC03-1、HLC01-1、HLC02-1、HLC05-3 |
| TypeE | **.** | **.** | **.** | **.** | **.** | **.** | SNC01-1、SNC02-1、SNC03-1、SXC09-1、SXC09-2、SXC09-3、SXC10-1、SXC11-1、SXC12-1、SXC01-1、SXC02-1、SXC03-1、SXC05-1、SXC06-1 |
| TypeF | **.** | **.** | **.** | **.** | **.** | **.** | SNC07-1、SNC07-2、SNC07-3、SNC08-1、SNC08-2、SNC08-3、SNC09-1、SNC09-2、SNC09-3、SNC11-1、SNC11-2、SNC11-3、SNC13-1、SNC13-2、SNC13-3 |
| TypeD | **.** | A | **.** | **.** | **.** | **.** | GSC12-1、SNW01-2 |
| TypeD | **.** | **.** | **.** | **.** | C | **.** | GSC13-1 |
| TypeF | **.** | **.** | **.** | **.** | C | **.** | SNC10-2、SNC10-3 |
| TypeB | **.** | **.** | T | **.** | **.** | **.** | HLC03-1、HLC04-3、HLC06-1 |
| *B. chinense* | **.** | **.** | T | **.** | **.** | **.** | SNW03-2 |
| TypeC | **.** | **.** | **.** | G | **.** | C | GSC03-1、GSC01-1、GSC02-1、GSC04-1、GSC05-1、GSC05-2 |
| *B. scorzonerifolium* | **.** | **.** | T | **.** | C | **.** | HEW01-1 |
| *B. chinense* | **.** | **.** | T | **.** | C | **.** | SXW01-2 |
