# Supplementary material for: Identification of the original plants of cultivated Bupleuri Radix based on DNA barcoding and chloroplast genome analysis
Source: PeerJ. 2022 Apr 12;10:e13208. doi: 10.7717/peerj.13208 (PMC9012172; doi:10.7717/peerj.13208)
Supplement: Supplemental Information 19 [file peerj-10-13208-s019.docx]

| **Similarity** | **Type C**  **(GSC03-1)** | **Type A1**  **(HLC05-3)** | **Type B**  **(HLC04-3)** | **Type A2**  **(HEC02-3)** | **Type E**  **(SXC04-1)** | **Type F**  **(SNC10-1)** | **Type D**  **(GSC06-1)** |
| --- | --- | --- | --- | --- | --- | --- | --- |
| Type C(GSC03-1) |  | 96.91% | 96.81% | 96.89% | 96.77% | 96.72% | 96.73% |
| Type A1(HLC05-3) |  |  | 98.91% | 99.86% | 99.02% | 98.96% | 98.99% |
| Type B(HLC04-3) |  |  |  | 98.89% | 98.65% | 98.58% | 98.63% |
| Type A2(HEC02-3) |  |  |  |  | 99.00% | 98.93% | 98.98% |
| Type E(SXC04-1) |  |  |  |  |  | 99.70% | 99.75% |
| Type F(SNC10-1) |  |  |  |  |  |  | 99.64% |
| Type D(GSC06-1) |  |  |  |  |  |  |  |
