# Supplementary material for: Identification of the original plants of cultivated Bupleuri Radix based on DNA barcoding and chloroplast genome analysis
Source: PeerJ. 2022 Apr 12;10:e13208. doi: 10.7717/peerj.13208 (PMC9012172; doi:10.7717/peerj.13208)
Supplement: Supplemental Information 20 [file peerj-10-13208-s020.docx]

| Species name | B. scorzonerifolium MT075715 | B. scorzonerifolium MT239475 | B. chinense MT075710 | B. chinense MT075713 | B. chinense MN893666 | B. chinense MT075709 | B. chinense NC 046774 | B. falcatum MT075716 | B. falcatum MT075714 | B. falcatum MT821947 | B. falcatum NC 027834 | B. latissimum MT821949 | B. latissimum NC 033346 | B. marginatum var. stenophyllum MT075712 | B. marginatum MN968501 |
| --- | --- | --- | --- | --- | --- | --- | --- | --- | --- | --- | --- | --- | --- | --- | --- |
| B. scorzonerifolium MT075715 |  |  |  |  |  |  |  |  |  |  |  |  |  |  |  |
| B. scorzonerifolium MT239475 | 0.00061 |  |  |  |  |  |  |  |  |  |  |  |  |  |  |
| B. chinense MT075710 | 0.00526 | 0.00522 |  |  |  |  |  |  |  |  |  |  |  |  |  |
| B. chinense MT075713 | 0.00516 | 0.00512 | 0.00071 |  |  |  |  |  |  |  |  |  |  |  |  |
| B. chinense MN893666 | 0.00510 | 0.00506 | 0.00043 | 0.00061 |  |  |  |  |  |  |  |  |  |  |  |
| B. chinense MT075709 | 0.00526 | 0.00521 | 0.00071 | 0.00082 | 0.00050 |  |  |  |  |  |  |  |  |  |  |
| B. chinense NC 046774 | 0.00525 | 0.00521 | 0.00075 | 0.00088 | 0.00053 | 0.00061 |  |  |  |  |  |  |  |  |  |
| B. falcatum MT075716 | 0.00361 | 0.00356 | 0.00306 | 0.00299 | 0.00285 | 0.00304 | 0.00307 |  |  |  |  |  |  |  |  |
| B. falcatum MT075714 | 0.00357 | 0.00351 | 0.00307 | 0.00300 | 0.00311 | 0.00304 | 0.00306 | 0.00026 |  |  |  |  |  |  |  |
| B. falcatum MT821947 | 0.00356 | 0.00350 | 0.00306 | 0.00299 | 0.00311 | 0.00302 | 0.00304 | 0.00025 | 0.00001 |  |  |  |  |  |  |
| B. falcatum NC 027834 | 0.00356 | 0.00350 | 0.00305 | 0.00297 | 0.00309 | 0.00301 | 0.00304 | 0.00025 | 0.00022 | 0.00021 |  |  |  |  |  |
| B. latissimum MT821949 | 0.00437 | 0.00432 | 0.00404 | 0.00409 | 0.00408 | 0.00418 | 0.00422 | 0.00276 | 0.00274 | 0.00272 | 0.00270 |  |  |  |  |
| B. latissimum NC 033346 | 0.00437 | 0.00432 | 0.00403 | 0.00409 | 0.00409 | 0.00417 | 0.00421 | 0.00274 | 0.00272 | 0.00271 | 0.00269 | 0.00008 |  |  |  |
| B. marginatum var. stenophyllum MT075712 | 0.01306 | 0.01302 | 0.01388 | 0.01393 | 0.01385 | 0.01399 | 0.01409 | 0.01273 | 0.01270 | 0.01270 | 0.01269 | 0.01333 | 0.01331 |  |  |
| B. marginatum MN968501 | 0.01341 | 0.01343 | 0.01427 | 0.01434 | 0.01425 | 0.01441 | 0.01453 | 0.01312 | 0.01309 | 0.01308 | 0.01309 | 0.01359 | 0.01357 | 0.00559 |  |
