# Supplementary material for: Identification of the original plants of cultivated Bupleuri Radix based on DNA barcoding and chloroplast genome analysis
Source: PeerJ. 2022 Apr 12;10:e13208. doi: 10.7717/peerj.13208 (PMC9012172; doi:10.7717/peerj.13208)
Supplement: Supplemental Information 21 [file peerj-10-13208-s021.docx]

| **No.** | **Gene** | **Length** | **S** | **Eta** | **Pi** | | **Theta** |
| --- | --- | --- | --- | --- | --- | --- | --- |
| 1 | Start_trnH | 4 | 1 | 1 | | 0.142857 | 0.102041 |
| 2 | atpF-exon1_atpH | 399 | 46 | 47 | | 0.045829 | 0.048079 |
| 3 | psbH_petB-exon1 | 119 | 9 | 10 | | 0.034014 | 0.034299 |
| 4 | ccsA_ndhD | 213 | 20 | 21 | | 0.029734 | 0.040241 |
| 5 | rpoA_rps11 | 79 | 6 | 7 | | 0.027125 | 0.036166 |
| 6 | rpl36_infA | 109 | 9 | 9 | | 0.026212 | 0.033702 |
| 7 | ndhE_ndhG | 203 | 13 | 15 | | 0.025803 | 0.03016 |
| 8 | rpl22_rps19 | 80 | 7 | 7 | | 0.025 | 0.035714 |
| 9 | trnR_atpA | 111 | 7 | 7 | | 0.024882 | 0.02574 |
| 10 | ndhI_ndhA-exon2 | 93 | 6 | 6 | | 0.023554 | 0.026333 |
| 11 | rps2_rpoC2 | 272 | 17 | 18 | | 0.022759 | 0.027011 |
| 12 | ndhD_psaC | 113 | 5 | 5 | | 0.022756 | 0.01806 |
| 13 | rps18_rpl20 | 231 | 13 | 13 | | 0.022264 | 0.02297 |
| 14 | trnP_psaJ | 417 | 30 | 30 | | 0.022154 | 0.029364 |
| 15 | trnV-exon1_trnM | 175 | 10 | 10 | | 0.021225 | 0.023324 |
| 16 | rps15_ycf1 | 398 | 24 | 25 | | 0.021177 | 0.025638 |
| 17 | petN_psbM | 962 | 53 | 53 | | 0.020691 | 0.022487 |
| 18 | rpl32_trnL | 665 | 38 | 39 | | 0.019835 | 0.023937 |
| 19 | trnK-exon1_rps16-exon2 | 761 | 44 | 44 | | 0.019148 | 0.0236 |
| 20 | ycf4_cemA | 548 | 31 | 31 | | 0.018422 | 0.02309 |
| 21 | petD-exon2_rpoA | 121 | 7 | 7 | | 0.018103 | 0.023613 |
| 22 | ndhF_rpl32 | 1203 | 64 | 65 | | 0.017496 | 0.022054 |
| 23 | petA_psbJ | 981 | 45 | 45 | | 0.017087 | 0.018723 |
| 24 | ndhG_ndhI | 323 | 15 | 15 | | 0.016807 | 0.018955 |
| 25 | rps16-exon1_trnQ | 1158 | 60 | 61 | | 0.016737 | 0.021501 |
| 26 | rpl16-exon1_rps3 | 149 | 8 | 8 | | 0.016619 | 0.021915 |
| 27 | trnS_rps4 | 319 | 17 | 17 | | 0.016122 | 0.021752 |
| 28 | psaI_ycf4 | 386 | 16 | 18 | | 0.016038 | 0.019034 |
| 29 | rps8_rpl14 | 202 | 10 | 10 | | 0.01603 | 0.020206 |
| 30 | ndhC_trnV-exon2 | 1158 | 54 | 55 | | 0.015996 | 0.019386 |
| 31 | psaA_ycf3-exon3 | 472 | 20 | 20 | | 0.01594 | 0.017295 |
| 32 | trnD_trnY | 110 | 5 | 5 | | 0.015584 | 0.018553 |
| 33 | trnL-exon2_trnF | 346 | 14 | 16 | | 0.015552 | 0.018875 |
| 34 | psaJ_rpl33 | 440 | 19 | 19 | | 0.015368 | 0.017625 |
| 35 | psbB_psbT | 199 | 9 | 9 | | 0.015315 | 0.01846 |
| 36 | rpl14_rpl16-exon2 | 132 | 4 | 4 | | 0.015152 | 0.012369 |
| 37 | trnH_psbA | 305 | 15 | 15 | | 0.014988 | 0.020074 |
| 38 | cemA_petA | 248 | 11 | 12 | | 0.014785 | 0.01975 |
| 39 | accD_psaI | 696 | 28 | 28 | | 0.013957 | 0.01642 |
| 40 | atpH_atpI | 771 | 30 | 30 | | 0.013835 | 0.015882 |
| 41 | trnS_trnG-exon1 | 697 | 27 | 27 | | 0.013391 | 0.015811 |
| 42 | trnG_trnfM | 171 | 6 | 6 | | 0.013367 | 0.014322 |
| 43 | trnE_trnT | 565 | 23 | 23 | | 0.013148 | 0.016616 |
| 44 | trnL_ccsA | 87 | 3 | 3 | | 0.013136 | 0.014075 |
| 45 | rpoB_trnC | 1190 | 43 | 44 | | 0.012565 | 0.015092 |
| 46 | ndhA-exon2_ndhA-exon1 | 1076 | 36 | 38 | | 0.012436 | 0.014415 |
| 47 | psbK_psbI | 423 | 16 | 16 | | 0.012383 | 0.015439 |
| 48 | trnM_atpE | 208 | 8 | 8 | | 0.012363 | 0.015699 |
| 49 | clpP-exon1_psbB | 439 | 16 | 17 | | 0.012257 | 0.015806 |
| 50 | psbI_trnS | 143 | 6 | 6 | | 0.011988 | 0.017126 |
| 51 | atpI_rps2 | 264 | 11 | 11 | | 0.011905 | 0.017007 |
| 52 | rbcL_accD | 697 | 25 | 25 | | 0.011614 | 0.01464 |
| 53 | rps4_trnT | 351 | 14 | 14 | | 0.011396 | 0.01628 |
| 54 | trnC_petN | 672 | 26 | 26 | | 0.011338 | 0.015792 |
| 55 | trnF_ndhJ | 354 | 12 | 12 | | 0.01103 | 0.013836 |
| 56 | trnG-exon2_trnR | 210 | 7 | 7 | | 0.010884 | 0.013605 |
| 57 | atpA_atpF-exon2 | 53 | 2 | 2 | | 0.010782 | 0.015402 |
| 58 | rpl16-exon2_rpl16-exon1 | 910 | 25 | 25 | | 0.01078 | 0.011213 |
| 59 | trnT_trnL-exon1 | 685 | 22 | 22 | | 0.010706 | 0.013109 |
| 60 | petG_trnW | 141 | 5 | 5 | | 0.010132 | 0.014474 |
| 61 | trnQ_psbK | 350 | 10 | 10 | | 0.009796 | 0.011662 |
| 62 | clpP-exon3_clpP-exon2 | 609 | 15 | 15 | | 0.009539 | 0.010053 |
| 63 | psbZ_trnG | 294 | 7 | 7 | | 0.009394 | 0.009718 |
| 64 | trnT_psbD | 1170 | 33 | 35 | | 0.009361 | 0.01221 |
| 65 | psbC_trnS | 238 | 7 | 7 | | 0.009204 | 0.012005 |
| 66 | clpP-exon2_clpP-exon1 | 845 | 25 | 25 | | 0.008904 | 0.012076 |
| 67 | matK_trnK-exon1 | 723 | 19 | 19 | | 0.008826 | 0.010726 |
| 68 | rpl33_rps18 | 164 | 4 | 5 | | 0.00842 | 0.012444 |
| 69 | petB-exon2_petD-exon1 | 175 | 5 | 5 | | 0.008163 | 0.011662 |
| 70 | trnK-exon2_matK | 284 | 8 | 8 | | 0.008048 | 0.011498 |
| 71 | petD-exon1_petD-exon2 | 746 | 18 | 18 | | 0.007788 | 0.009848 |
| 72 | rps16-exon2_rps16-exon1 | 870 | 23 | 23 | | 0.007772 | 0.010791 |
| 73 | trnG-exon1_trnG-exon2 | 696 | 17 | 17 | | 0.007526 | 0.00997 |
| 74 | psbM_trnD | 692 | 15 | 16 | | 0.007363 | 0.009437 |
| 75 | ycf3-exon1_trnS | 591 | 13 | 13 | | 0.006929 | 0.008978 |
| 76 | psbN_psbH | 84 | 2 | 2 | | 0.006803 | 0.009718 |
| 77 | psaC_ndhE | 254 | 6 | 6 | | 0.006749 | 0.009642 |
| 78 | petB-exon1_petB-exon2 | 727 | 16 | 16 | | 0.006681 | 0.008983 |
| 79 | rpoC1-exon2_rpoC1-exon1 | 740 | 15 | 15 | | 0.006564 | 0.008274 |
| 80 | ycf3-exon3_ycf3-exon2 | 771 | 17 | 17 | | 0.006547 | 0.009 |
| 81 | rpl20_rps12-exon1 | 690 | 15 | 15 | | 0.006487 | 0.008873 |
| 82 | trnL-exon1_trnL-exon2 | 489 | 10 | 10 | | 0.006427 | 0.008347 |
| 83 | atpB_rbcL | 758 | 15 | 15 | | 0.006408 | 0.008077 |
| 84 | psbE_petL | 880 | 18 | 18 | | 0.006277 | 0.008349 |
| 85 | rps12-exon1_clpP-exon3 | 137 | 3 | 3 | | 0.006257 | 0.008938 |
| 86 | ycf3-exon2_ycf3-exon1 | 714 | 13 | 13 | | 0.005869 | 0.007432 |
| 87 | atpF-exon2_atpF-exon1 | 633 | 11 | 11 | | 0.005868 | 0.007093 |
| 88 | ndhK_ndhC | 49 | 1 | 1 | | 0.005831 | 0.00833 |
| 89 | trnS_psbZ | 285 | 4 | 4 | | 0.005681 | 0.005729 |
| 90 | petL_petG | 152 | 2 | 2 | | 0.005639 | 0.005371 |
| 91 | trnfM_rps14 | 154 | 3 | 3 | | 0.005566 | 0.007951 |
| 92 | ndhJ_ndhK | 105 | 1 | 1 | | 0.005442 | 0.003887 |
| 93 | rpoC2_rpoC1-exon2 | 217 | 3 | 3 | | 0.005267 | 0.005643 |
| 94 | psbT_psbN | 91 | 1 | 1 | | 0.005233 | 0.004485 |
| 95 | rps19_rpl2-exon2 | 57 | 1 | 1 | | 0.005013 | 0.007161 |
| 96 | infA_rps8 | 121 | 2 | 2 | | 0.004723 | 0.006747 |
| 97 | rpl2-exon2_ | 127 | 2 | 2 | | 0.004499 | 0.006428 |
| 98 | rrn5_trnR | 256 | 3 | 3 | | 0.004464 | 0.004783 |
| 99 | trnR_rrn5 | 256 | 3 | 3 | | 0.004464 | 0.004783 |
| 100 | psbJ_psbL | 132 | 2 | 2 | | 0.004329 | 0.006184 |
| 101 | trnW_trnP | 134 | 2 | 2 | | 0.004264 | 0.006092 |
| 102 | rrn16_trnI-exon1 | 297 | 3 | 3 | | 0.003848 | 0.004123 |
| 103 | trnI-exon1_rrn16 | 297 | 3 | 3 | | 0.003848 | 0.004123 |
| 104 | ndhH_rps15 | 91 | 1 | 1 | | 0.00314 | 0.004485 |
| 105 | rps11_rpl36 | 95 | 1 | 1 | | 0.003008 | 0.004297 |
| 106 | psbA_trnK-exon2 | 213 | 2 | 2 | | 0.002683 | 0.003833 |
| 107 | ndhB-exon2_ndhB-exon1 | 682 | 5 | 5 | | 0.002653 | 0.002992 |
| 108 | ndhB-exon1_ndhB-exon2 | 682 | 5 | 5 | | 0.002653 | 0.002992 |
| 109 | trnN_ndhF | 2219 | 19 | 19 | | 0.002618 | 0.003495 |
| 110 | rps12-exon2_trnV | 1826 | 12 | 12 | | 0.002347 | 0.002682 |
| 111 | ycf2_trnL | 624 | 4 | 4 | | 0.002289 | 0.002616 |
| 112 | trnL_ycf2 | 624 | 4 | 4 | | 0.002289 | 0.002616 |
| 113 | trnV-exon2_trnV-exon1 | 559 | 4 | 4 | | 0.002045 | 0.002921 |
| 114 | ndhB-exon1_rps7 | 298 | 1 | 1 | | 0.001918 | 0.00137 |
| 115 | rps7_ndhB-exon1 | 298 | 1 | 1 | | 0.001918 | 0.00137 |
| 116 | trnV_rps12-exon2 | 1825 | 10 | 10 | | 0.001879 | 0.002237 |
| 117 | trnR_trnN | 578 | 3 | 3 | | 0.001813 | 0.002119 |
| 118 | trnN_trnR | 578 | 3 | 3 | | 0.001813 | 0.002119 |
| 119 | ycf1_trnN | 327 | 2 | 2 | | 0.001748 | 0.002496 |
| 120 | rpl23_trnI | 165 | 1 | 1 | | 0.001732 | 0.002474 |
| 121 | trnI_rpl23 | 165 | 1 | 1 | | 0.001732 | 0.002474 |
| 122 | trnA-exon1_trnA-exon2 | 813 | 4 | 4 | | 0.001406 | 0.002008 |
| 123 | trnA-exon2_trnA-exon1 | 813 | 4 | 4 | | 0.001406 | 0.002008 |
| 124 | trnL_ndhB-exon2 | 564 | 2 | 2 | | 0.001351 | 0.001447 |
| 125 | ndhB-exon2_trnL | 564 | 2 | 2 | | 0.001351 | 0.001447 |
| 126 | trnV_rrn16 | 227 | 1 | 1 | | 0.001259 | 0.001798 |
| 127 | rrn16_trnV | 227 | 1 | 1 | | 0.001259 | 0.001798 |
| 128 | trnI-exon1_trnI-exon2 | 943 | 4 | 4 | | 0.001212 | 0.001731 |
| 129 | trnI-exon2_trnI-exon1 | 943 | 4 | 4 | | 0.001212 | 0.001731 |
| 130 | rpl2-exon2_rpl2-exon1 | 648 | 2 | 2 | | 0.001176 | 0.00126 |
| 131 | rpl2-exon1_rpl2-exon2 | 648 | 2 | 2 | | 0.001176 | 0.00126 |
| 132 | rpoC1-exon1_rpoB | 5 | 0 | 0 | | 0 | 0 |
| 133 | trnY_trnE | 85 | 0 | 0 | | 0 | 0 |
| 134 | psbD_psbC | 57 | 0 | 0 | | 0 | 0 |
| 135 | rps14_psaB | 128 | 0 | 0 | | 0 | 0 |
| 136 | psaB_psaA | 25 | 0 | 0 | | 0 | 0 |
| 137 | atpE_atpB | 8 | 0 | 0 | | 0 | 0 |
| 138 | psbL_psbF | 22 | 0 | 0 | | 0 | 0 |
| 139 | psbF_psbE | 9 | 0 | 0 | | 0 | 0 |
| 140 | rps12-exon1_rps12-exon1 | 538 | 0 | 0 | | 0 | 0 |
| 141 | rps3_rpl22 | 20 | 0 | 0 | | 0 | 0 |
| 142 | rpl2-exon1_rpl23 | 18 | 0 | 0 | | 0 | 0 |
| 143 | trnI_ycf2 | 88 | 0 | 0 | | 0 | 0 |
| 144 | rps7_rps12-exon3 | 55 | 0 | 0 | | 0 | 0 |
| 145 | rps12-exon3_rps12-exon2 | 536 | 0 | 0 | | 0 | 0 |
| 146 | trnI-exon2_trnA-exon1 | 64 | 0 | 0 | | 0 | 0 |
| 147 | trnA-exon2_rrn23 | 152 | 0 | 0 | | 0 | 0 |
| 148 | rrn23_rrn4.5 | 98 | 0 | 0 | | 0 | 0 |
| 149 | rrn4.5_rrn5 | 248 | 0 | 0 | | 0 | 0 |
| 150 | ndhA-exon1_ndhH | 1 | 0 | 0 | | 0 | 0 |
| 151 | rrn5_rrn4.5 | 248 | 0 | 0 | | 0 | 0 |
| 152 | rrn4.5_rrn23 | 98 | 0 | 0 | | 0 | 0 |
| 153 | rrn23_trnA-exon2 | 152 | 0 | 0 | | 0 | 0 |
| 154 | trnA-exon1_trnI-exon2 | 64 | 0 | 0 | | 0 | 0 |
| 155 | rps12-exon2_rps12-exon3 | 536 | 0 | 0 | | 0 | 0 |
| 156 | rps12-exon3_rps7 | 55 | 0 | 0 | | 0 | 0 |
| 157 | ycf2_trnI | 88 | 0 | 0 | | 0 | 0 |
| 158 | rpl23_rpl2-exon1 | 18 | 0 | 0 | | 0 | 0 |
