# Supplementary material for: Identification of the original plants of cultivated Bupleuri Radix based on DNA barcoding and chloroplast genome analysis
Source: PeerJ. 2022 Apr 12;10:e13208. doi: 10.7717/peerj.13208 (PMC9012172; doi:10.7717/peerj.13208)
Supplement: Supplemental Information 22 [file peerj-10-13208-s022.docx]

| **No.** | **Gene** | **Length** | **S** | **Eta** | **Pi** | **Theta** |
| --- | --- | --- | --- | --- | --- | --- |
| 1 | ycf1 | 5481 | 173 | 177 | 0.010287 | 0.013181 |
| 2 | petL | 96 | 3 | 3 | 0.008929 | 0.012755 |
| 3 | rpl33 | 201 | 5 | 5 | 0.008529 | 0.010153 |
| 4 | ndhF | 2250 | 51 | 51 | 0.008085 | 0.009252 |
| 5 | matK | 1515 | 35 | 35 | 0.007984 | 0.00943 |
| 6 | ndhK | 678 | 14 | 14 | 0.007866 | 0.008428 |
| 7 | trnK-exon1 | 37 | 1 | 1 | 0.007722 | 0.011031 |
| 8 | atpI | 744 | 16 | 16 | 0.007297 | 0.008778 |
| 9 | rps15 | 273 | 6 | 6 | 0.006977 | 0.008971 |
| 10 | rps8 | 399 | 9 | 9 | 0.006922 | 0.009207 |
| 11 | psaJ | 141 | 2 | 2 | 0.006755 | 0.00579 |
| 12 | cemA | 693 | 13 | 13 | 0.006459 | 0.007657 |
| 13 | ndhH | 1182 | 21 | 22 | 0.006285 | 0.007597 |
| 14 | accD | 1470 | 24 | 25 | 0.00609 | 0.006942 |
| 15 | rpoC2 | 4122 | 73 | 73 | 0.005938 | 0.007229 |
| 16 | ccsA | 963 | 17 | 17 | 0.005439 | 0.007205 |
| 17 | ndhA-exon2 | 539 | 10 | 10 | 0.005301 | 0.007573 |
| 18 | rpl14 | 369 | 6 | 6 | 0.005162 | 0.006637 |
| 19 | rpl36 | 114 | 2 | 2 | 0.005013 | 0.007161 |
| 20 | rps3 | 657 | 10 | 10 | 0.004929 | 0.006213 |
| 21 | infA | 234 | 3 | 3 | 0.004884 | 0.005233 |
| 22 | rpl16-exon2 | 399 | 5 | 5 | 0.004774 | 0.005115 |
| 23 | ndhC | 363 | 6 | 6 | 0.004723 | 0.006747 |
| 24 | ndhE | 306 | 4 | 4 | 0.004669 | 0.005336 |
| 25 | atpF-exon2 | 462 | 7 | 7 | 0.004329 | 0.006184 |
| 26 | ndhD | 1503 | 18 | 18 | 0.004246 | 0.004888 |
| 27 | rpoB | 3213 | 38 | 39 | 0.004224 | 0.004954 |
| 28 | psbK | 186 | 2 | 2 | 0.004096 | 0.004389 |
| 29 | trnG | 71 | 1 | 1 | 0.004024 | 0.005749 |
| 30 | clpP-exon1 | 71 | 1 | 1 | 0.004024 | 0.005749 |
| 31 | trnQ | 72 | 1 | 1 | 0.003968 | 0.005669 |
| 32 | ycf4 | 555 | 7 | 7 | 0.003947 | 0.005148 |
| 33 | clpP-exon2 | 292 | 4 | 4 | 0.003914 | 0.005591 |
| 34 | rps2 | 711 | 9 | 9 | 0.003885 | 0.005167 |
| 35 | trnW | 74 | 1 | 1 | 0.003861 | 0.005516 |
| 36 | ycf3-exon3 | 153 | 2 | 2 | 0.003735 | 0.005336 |
| 37 | rpoA | 1074 | 11 | 12 | 0.003591 | 0.004561 |
| 38 | atpB | 1495 | 14 | 14 | 0.003567 | 0.003822 |
| 39 | rps16-exon2 | 241 | 2 | 2 | 0.003557 | 0.003387 |
| 40 | rpoC1-exon2 | 1605 | 16 | 16 | 0.003501 | 0.004069 |
| 41 | rpl20 | 387 | 4 | 4 | 0.003445 | 0.004219 |
| 42 | atpE | 402 | 3 | 3 | 0.003317 | 0.003046 |
| 43 | rbcL | 1428 | 14 | 14 | 0.003135 | 0.004002 |
| 44 | psaA | 2253 | 20 | 20 | 0.003086 | 0.003623 |
| 45 | rps19 | 279 | 3 | 3 | 0.003072 | 0.004389 |
| 46 | psbZ | 189 | 2 | 2 | 0.003023 | 0.004319 |
| 47 | petB-exon2 | 642 | 6 | 6 | 0.002967 | 0.003815 |
| 48 | rpoC1-exon1 | 453 | 3 | 3 | 0.002943 | 0.002703 |
| 49 | ndhA-exon1 | 553 | 5 | 5 | 0.002928 | 0.00369 |
| 50 | rpl32 | 165 | 1 | 1 | 0.002886 | 0.002474 |
| 51 | atpA | 1524 | 13 | 13 | 0.002875 | 0.003482 |
| 52 | ndhI | 504 | 5 | 5 | 0.002835 | 0.004049 |
| 53 | rps18 | 306 | 3 | 3 | 0.002801 | 0.004002 |
| 54 | psbI | 111 | 1 | 1 | 0.002574 | 0.003677 |
| 55 | rps12-exon1 | 114 | 1 | 1 | 0.002506 | 0.00358 |
| 56 | rps12-exon1 | 114 | 1 | 1 | 0.002506 | 0.00358 |
| 57 | ycf3-exon2 | 229 | 1 | 1 | 0.002495 | 0.001782 |
| 58 | psbA | 1062 | 7 | 7 | 0.002421 | 0.00269 |
| 59 | petD-exon2 | 475 | 4 | 4 | 0.002406 | 0.003437 |
| 60 | rpl22 | 480 | 4 | 4 | 0.002381 | 0.003401 |
| 61 | psbB | 1527 | 10 | 10 | 0.00237 | 0.002673 |
| 62 | ycf3-exon1 | 125 | 1 | 1 | 0.002286 | 0.003265 |
| 63 | petA | 963 | 6 | 6 | 0.002275 | 0.002543 |
| 64 | rps11 | 417 | 3 | 3 | 0.002056 | 0.002936 |
| 65 | psbC | 1422 | 10 | 10 | 0.002009 | 0.00287 |
| 66 | rps14 | 303 | 2 | 2 | 0.001886 | 0.002694 |
| 67 | ndhJ | 477 | 2 | 2 | 0.001797 | 0.001711 |
| 68 | psaB | 2205 | 12 | 12 | 0.001728 | 0.002221 |
| 69 | ycf2 | 6297 | 29 | 29 | 0.001573 | 0.00188 |
| 70 | ycf2 | 6297 | 29 | 29 | 0.001573 | 0.00188 |
| 71 | rps4 | 606 | 3 | 3 | 0.001414 | 0.002021 |
| 72 | clpP-exon3 | 231 | 1 | 1 | 0.001237 | 0.001767 |
| 73 | rps12-exon2 | 232 | 1 | 1 | 0.001232 | 0.001759 |
| 74 | rps12-exon2 | 232 | 1 | 1 | 0.001232 | 0.001759 |
| 75 | psbH | 240 | 1 | 1 | 0.001191 | 0.001701 |
| 76 | rpl2-exon2 | 435 | 1 | 1 | 0.001095 | 0.000938 |
| 77 | rpl2-exon2 | 435 | 1 | 1 | 0.001095 | 0.000938 |
| 78 | psbD | 1062 | 3 | 3 | 0.000807 | 0.001153 |
| 79 | ndhB-exon2 | 756 | 2 | 2 | 0.000756 | 0.00108 |
| 80 | ndhB-exon2 | 756 | 2 | 2 | 0.000756 | 0.00108 |
| 81 | rpl2-exon1 | 393 | 1 | 1 | 0.000727 | 0.001039 |
| 82 | rpl2-exon1 | 393 | 1 | 1 | 0.000727 | 0.001039 |
| 83 | ndhG | 531 | 1 | 1 | 0.000538 | 0.000769 |
| 84 | ndhB-exon1 | 777 | 1 | 1 | 0.000368 | 0.000525 |
| 85 | ndhB-exon1 | 777 | 1 | 1 | 0.000368 | 0.000525 |
| 86 | rrn23 | 2810 | 2 | 2 | 0.000203 | 0.000291 |
| 87 | rrn23 | 2810 | 2 | 2 | 0.000203 | 0.000291 |
| 88 | rrn16 | 1491 | 1 | 1 | 0.000192 | 0.000274 |
| 89 | rrn16 | 1491 | 1 | 1 | 0.000192 | 0.000274 |
| 90 | trnH | 74 | 0 | 0 | 0 | 0 |
| 91 | trnK-exon2 | 35 | 0 | 0 | 0 | 0 |
| 92 | rps16-exon1 | 41 | 0 | 0 | 0 | 0 |
| 93 | trnS | 88 | 0 | 0 | 0 | 0 |
| 94 | trnG-exon1 | 23 | 0 | 0 | 0 | 0 |
| 95 | trnG-exon2 | 48 | 0 | 0 | 0 | 0 |
| 96 | trnR | 72 | 0 | 0 | 0 | 0 |
| 97 | atpF-exon1 | 159 | 0 | 0 | 0 | 0 |
| 98 | atpH | 246 | 0 | 0 | 0 | 0 |
| 99 | trnC | 81 | 0 | 0 | 0 | 0 |
| 100 | petN | 90 | 0 | 0 | 0 | 0 |
| 101 | psbM | 117 | 0 | 0 | 0 | 0 |
| 102 | trnD | 74 | 0 | 0 | 0 | 0 |
| 103 | trnY | 84 | 0 | 0 | 0 | 0 |
| 104 | trnE | 73 | 0 | 0 | 0 | 0 |
| 105 | trnT | 72 | 0 | 0 | 0 | 0 |
| 106 | trnS | 93 | 0 | 0 | 0 | 0 |
| 107 | trnfM | 74 | 0 | 0 | 0 | 0 |
| 108 | trnS | 86 | 0 | 0 | 0 | 0 |
| 109 | trnT | 73 | 0 | 0 | 0 | 0 |
| 110 | trnL-exon1 | 37 | 0 | 0 | 0 | 0 |
| 111 | trnL-exon2 | 49 | 0 | 0 | 0 | 0 |
| 112 | trnF | 73 | 0 | 0 | 0 | 0 |
| 113 | trnV-exon2 | 37 | 0 | 0 | 0 | 0 |
| 114 | trnV-exon1 | 39 | 0 | 0 | 0 | 0 |
| 115 | trnM | 73 | 0 | 0 | 0 | 0 |
| 116 | psaI | 111 | 0 | 0 | 0 | 0 |
| 117 | psbJ | 123 | 0 | 0 | 0 | 0 |
| 118 | psbL | 117 | 0 | 0 | 0 | 0 |
| 119 | psbF | 120 | 0 | 0 | 0 | 0 |
| 120 | psbE | 252 | 0 | 0 | 0 | 0 |
| 121 | petG | 114 | 0 | 0 | 0 | 0 |
| 122 | trnP | 75 | 0 | 0 | 0 | 0 |
| 123 | psbT | 111 | 0 | 0 | 0 | 0 |
| 124 | psbN | 132 | 0 | 0 | 0 | 0 |
| 125 | petB-exon1 | 6 | 0 | 0 | 0 | 0 |
| 126 | petD-exon1 | 8 | 0 | 0 | 0 | 0 |
| 127 | rpl16-exon1 | 9 | 0 | 0 | 0 | 0 |
| 128 | rpl23 | 282 | 0 | 0 | 0 | 0 |
| 129 | trnI | 74 | 0 | 0 | 0 | 0 |
| 130 | trnL | 81 | 0 | 0 | 0 | 0 |
| 131 | rps7 | 468 | 0 | 0 | 0 | 0 |
| 132 | rps12-exon3 | 26 | 0 | 0 | 0 | 0 |
| 133 | trnV | 72 | 0 | 0 | 0 | 0 |
| 134 | trnI-exon1 | 42 | 0 | 0 | 0 | 0 |
| 135 | trnI-exon2 | 35 | 0 | 0 | 0 | 0 |
| 136 | trnA-exon1 | 38 | 0 | 0 | 0 | 0 |
| 137 | trnA-exon2 | 35 | 0 | 0 | 0 | 0 |
| 138 | rrn4.5 | 103 | 0 | 0 | 0 | 0 |
| 139 | rrn5 | 121 | 0 | 0 | 0 | 0 |
| 140 | trnR | 74 | 0 | 0 | 0 | 0 |
| 141 | trnN | 72 | 0 | 0 | 0 | 0 |
| 142 | trnL | 80 | 0 | 0 | 0 | 0 |
| 143 | psaC | 246 | 0 | 0 | 0 | 0 |
| 144 | trnN | 72 | 0 | 0 | 0 | 0 |
| 145 | trnR | 74 | 0 | 0 | 0 | 0 |
| 146 | rrn5 | 121 | 0 | 0 | 0 | 0 |
| 147 | rrn4.5 | 103 | 0 | 0 | 0 | 0 |
| 148 | trnA-exon2 | 35 | 0 | 0 | 0 | 0 |
| 149 | trnA-exon1 | 38 | 0 | 0 | 0 | 0 |
| 150 | trnI-exon2 | 35 | 0 | 0 | 0 | 0 |
| 151 | trnI-exon1 | 42 | 0 | 0 | 0 | 0 |
| 152 | trnV | 72 | 0 | 0 | 0 | 0 |
| 153 | rps12-exon3 | 26 | 0 | 0 | 0 | 0 |
| 154 | rps7 | 468 | 0 | 0 | 0 | 0 |
| 155 | trnL | 81 | 0 | 0 | 0 | 0 |
| 156 | trnI | 74 | 0 | 0 | 0 | 0 |
| 157 | rpl23 | 282 | 0 | 0 | 0 | 0 |
